# Metformin strongly affects transcriptome of peripheral blood cells in healthy individuals

**DOI:** 10.1371/journal.pone.0224835

**Published:** 2019-11-08

**Authors:** Monta Briviba, Ivars Silamikelis, Ineta Kalnina, Laura Ansone, Vita Rovite, Ilze Elbere, Ilze Radovica-Spalvina, Davids Fridmanis, Jekaterina Aladyeva, Ilze Konrade, Valdis Pirags, Janis Klovins

**Affiliations:** 1 Latvian Biomedical Research and Study Centre, Riga, Latvia; 2 Riga Stradins University, Riga, Latvia; Consiglio Nazionale delle Ricerche, ITALY

## Abstract

Metformin is a commonly used antihyperglycaemic agent for the treatment of type 2 diabetes mellitus. Nevertheless, the exact mechanisms of action, underlying the various therapeutic effects of metformin, remain elusive. The goal of this study was to evaluate the alterations in longitudinal whole-blood transcriptome profiles of healthy individuals after a one-week metformin intervention in order to identify the novel molecular targets and further prompt the discovery of predictive biomarkers of metformin response. Next generation sequencing-based transcriptome analysis revealed metformin-induced differential expression of genes involved in intestinal immune network for IgA production and cytokine-cytokine receptor interaction pathways. Significantly elevated faecal sIgA levels during administration of metformin, and its correlation with the expression of genes associated with immune response (*CXCR4*, *HLA-DQA1*, *MAP3K14*, *TNFRSF21*, *CCL4*, *ACVR1B*, *PF4*, *EPOR*, *CXCL8*) supports a novel hypothesis of strong association between metformin and intestinal immune system, and for the first time provide evidence for altered RNA expression as a contributing mechanism of metformin’s action. In addition to universal effects, 4 clusters of functionally related genes with a subject-specific differential expression were distinguished, including genes relevant to insulin production (*HNF1B*, *HNF1A*, *HNF4A*, *GCK*, *INS*, *NEUROD1*, *PAX4*, *PDX1*, *ABCC8*, *KCNJ11*) and cholesterol homeostasis (*APOB*, *LDLR*, *PCSK9*). This inter-individual variation of the metformin effect on the transcriptional regulation goes in line with well-known variability of the therapeutic response to the drug.

## Introduction

Metformin is the first-line antidiabetic agent used in pharmacotherapy of type 2 diabetes to improve glucose homeostasis[1]. Various additional therapeutic benefits beyond its antihyperglycaemic action have been highlighted lately, justifying the pleiotropic effect of the drug. In patients with type 2 diabetes metformin therapy is associated with reduced cardiovascular morbidity[2]. In addition, metformin exposure has a protective role against tumorigenesis in various types of cancers and it is proved to be beneficial in the preventive oncology regardless of the diabetic state[3, 4]. Furthermore, metformin therapy is often recommended for women with polycystic ovary syndrome to improve insulin sensitivity, facilitate menstrual regularity, induce ovulation, reduce circulating androgen levels and body weight[5, 6]. Likewise, metformin application in the treatment of such neurodegenerative disorders as Alzheimer’s and Parkinson’s diseases is currently under consideration[7–9].

Several possible molecular mechanisms of metformin action have been proposed, including inhibition of mitochondrial respiratory-chain complex 1, the reduction of cyclic adenosine monophosphate (AMP) levels, activation of AMP-activated protein kinase (AMPK) and recently described interaction with gut microbiota[10–13]. Although they partially explain major beneficial effects of the drug, exact mechanisms of metformin action remain unclear even after 60 years since its first clinical use.

Despite the widespread application of metformin, approximately 30% of type 2 diabetes patients using the drug are failing to achieve the adequate glycemic control[14]. Moreover, 20%-30% of type 2 diabetes patients suffer metformin-associated gastrointestinal adverse events and about 5% discontinue the therapy because of severe intolerance[15, 16]. Heritability of the glycaemic response to metformin has been suggested to depend on many allelic variants with small to moderate effects[17]. Contribution of inheritance to variation in metformin response has gained great interest in the past years, encouraging numerous targeted studies investigating genes coding for organic cation transporters OCT1, OCT2, OCT3 and multidrug and toxin extrusion proteins MATE1 and MATE2-K[18–22]. Moreover, Genome-Wide Association Studies have revealed multiple genetic variations within *ATM*, *PRPF31*, *CPA6*, and *STAT* genes associated with metformin response[23–25]. However, genetic alterations explain only a small proportion of the heterogeneous response to metformin therapy, therefore omics-based investigation of the pleiotropic mechanism of the drug is needed to promote the development of biomarkers for therapeutic efficacy[26].

Previous studies have demonstrated metformin-mediated changes at the transcriptome level in various animal tissues, nevertheless studies of metformin-related transcriptome profiles in humans are lacking. For instance, recent study by Guo *et al*. discovered metformin-induced alterations of the coding transcriptome profile and non-coding RNAs in the liver of high-fat diet induced mouse model of non-alcoholic fatty liver disease[27]. Likewise, microarray analysis of mice liver and muscle tissues revealed the ability of metformin to mimic the calorie restriction-like transcriptome[28]. Furthermore, a distinct gene expression profile related to cardiovascular benefits of metformin, was observed in a rat model of obesity and insulin resistance[29]. Meanwhile, cell culture studies of adrenal H295R cell and MCF7 breast cancer cell transcriptome have revealed the association of metformin with complex cellular processes related with energy metabolism, steroidogenesis and the immune system as well as glycolysis and cancer-related pathways[30, 31].

To identify the genes targeted by metformin, we performed the whole-transcriptome analysis with total RNA sequencing on whole-blood samples, obtained from the healthy individuals undergoing a seven-day course of metformin. To the best of our knowledge, this is the first study providing information about the immediate effects of metformin administration on global gene expression in healthy individuals.

## Materials and methods

### Study design

**Fig 1:**
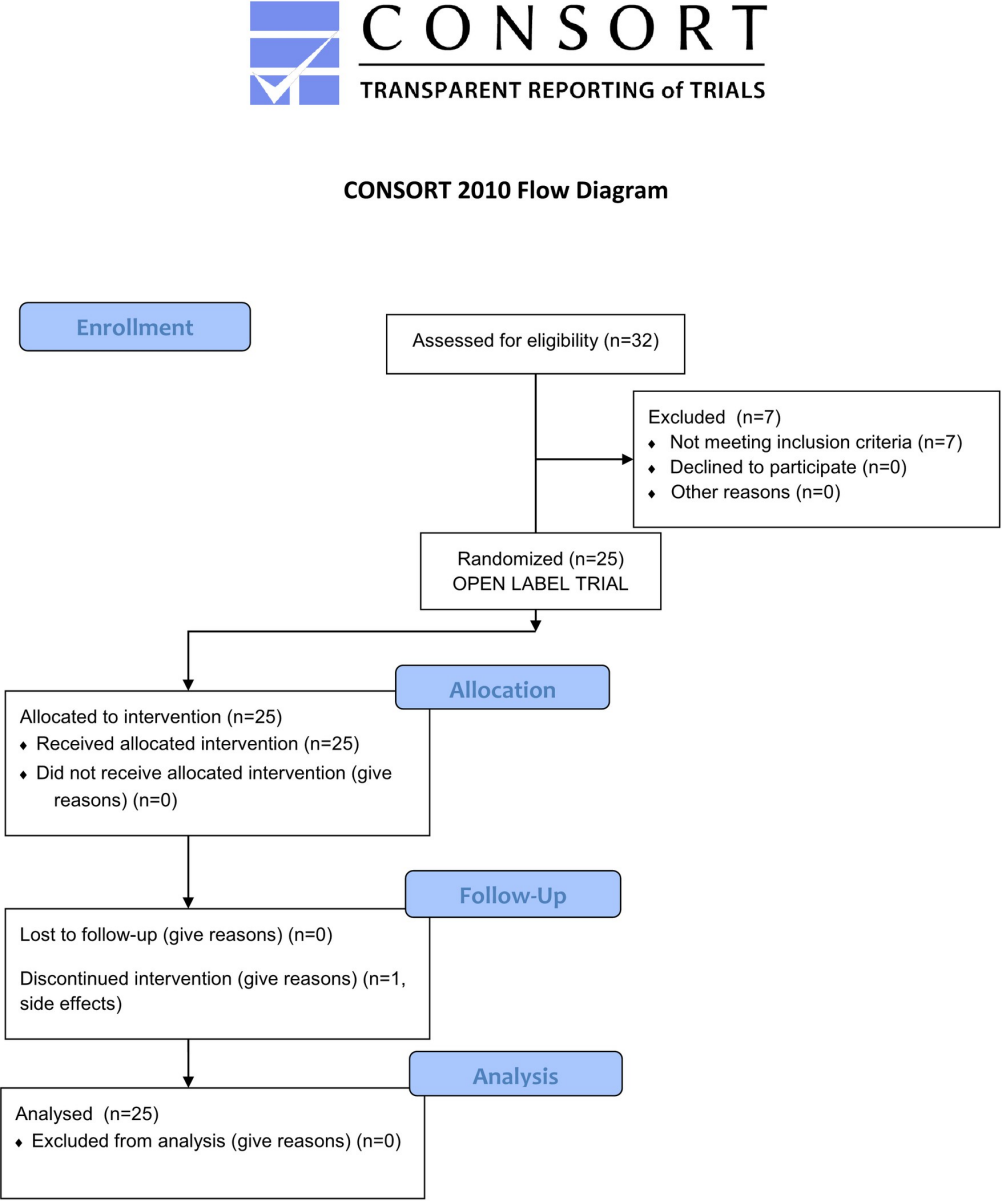
CONSORT flowchart of the open-label trial.

The study enrolled 25 healthy European descent volunteers with no history of chronic disease, meeting exclusion/inclusion criteria (S4 Table) set within the framework of the ongoing clinical trial ‘Pharmacodynamics of antidiabetic drug metformin’ (50 individuals to be included in total), protocol number MIKROMET16001, registration number of EU Clinical Trials Register: 2016-001092-74 (www.clinicaltrialsregister.eu) (Fig 1). Participants received twice-daily oral 850mg dose of metformin hydrochloride (*Metforal* 850mg, Berlin-Chemie AG) for 7 days. Medication adherence was reported by each participant at the end of the active period of the study. Fasting blood tests (e.g. measures of ALT, plasma glucose, creatinine levels) were performed in certified clinical laboratory 1–3 days before metformin administration in order to evaluate general hematological and biochemical parameters, and eligibility of volunteers (Table 1). RNA for transcriptome analysis was isolated from the whole blood samples collected in Latvian Biomedical Research and Study Centre at three time-points: (1) before administration of metformin (M0, morning, fasting state), 10 hours after the first metformin intake, but before the second dose (M10h, evening) and after 7 days long metformin course (M7d, morning, fasting state). The third blood sample was not collected from one out of 25 study subjects, due to the discontinuation of metformin treatment, nevertheless the rest of the blood samples collected from the particular subject were included in the data analysis. Longitudinal study design of the open-label trial was chosen as the most suitable method for global gene expression analysis with high inter-individual variability expected.

**Table 1:** Characteristics of the study group.

Characteristic	Value
Female/ male, n (%)	18 (72.0%) / 7 (28.0%)
Mean age (years) ± SD	34.4 ± 10.8
Mean BMI ± SD	25.5 ± 3.1
Mean ALAT ± SD, U/l	24.8 ± 11.9
Mean creatinine ± SD, μmol/l	66.2 ± 9.0
Fasting plasma glucose (mmol/l), mean ± SD	5.2 ± 0.4

BMI—body mass index; SD—standard deviation; ALAT—alanine aminotransferase

All individuals were concurrently involved in an ongoing analysis of gut microbiome and DNA methylation profiles according to the study protocol (S1 Text). The primary endpoint of the study was significantly shifted composition of the gut microbiome after administration of metformin. The secondary endpoint of the study was alterations in DNA methylation profiles and mRNA levels following metformin use.

Written informed consent was obtained from every participant and the study protocol was approved by the Central Medical Ethics committee of Latvia (1/16-05-12) and the State Agency of Medicines of the Republic of Latvia (17–1723). The research was conducted in accordance with the The Code of Ethics of the World Medical Association (Declaration of Helsinki) and International Conference on Harmonisation E6 (R2) Guidelines for Good Clinical Practice. All participants were included in the Genome Database of the Latvian Population[32].

### RNA sample preparation and next generation sequencing

Venous blood samples (n = 74) were collected in Tempus Blood RNA Tubes, followed by total RNA isolation with PerfectPure RNA Blood Kit (5Prime GmbH, Germany), according to the manufacturer's instructions. The integrity of the extracted RNA was evaluated by RNA integrity number (RIN) within Agilent 2100 Bioanalyzer system (Agilent, USA). Ribosomal RNA depletion was done with Low Input RiboMinus Eukaryote System v2 (Thermo Fisher Scientific, USA) by processing 500ng of total RNA from each sample. For cDNA library preparation we used Ion Total RNA-Seq Kit v2 (Thermo Fisher Scientific, USA), sequencing was done on Ion Proton System and Ion PI Chip (Thermo Fisher Scientific, USA), following the manufacturer’s instructions. Since shot-gun RNA sequencing is considered to be the most accurate and desirable method for quantification of expression of individual transcripts and genes, additional methods for technical validation were not applied in this study[33].

### Stool sample collection and detection of secretory IgA by ELISA

Within the framework of the clinical trial two aliquots of stool samples were collected from each study participant at three time points: before administration of metformin (M0), 24 hours after the first dose (M24h) and 7 days after the first intake of metformin (M7d), except for two participants for whom the third stool sample was not available (n = 73 in total). The samples were transferred at −80°C within 24 hours since the collection. The concentration of secretory immunoglobulin A (sIgA) in 100mg of each stool sample was determined by ImmuChrom ELISA Kit (ImmuChrom GmbH, Germany), according to the manufacturer’s instructions, the absorbance was read at 450nm and 620nm as the reference wavelength.

### Bioinformatic analysis

The sequencing reads were trimmed with Trimmomatic 0.36 using window size 5 and quality threshold 10. After trimming reads had to have a minimum length of 30bp and average quality of 10 to be included in subsequent analyzes. Sequencing reads were mapped against human reference genome GRCh38 release 90 and read counts were calculated with STAR 2.5.3a. The obtained read counts were normalized using trimmed mean normalization implemented in Bioconductor package edgeR in R. Differentially expressed genes (DEGs) were estimated with two different methods. At first, likelihood ratio test with added observation weights was used to reduce the influence of outliers and to obtain a list of DEGs, where the counts per million (CPM) value had to be 1 or more in at least 24 samples for the gene to be included in the analysis (edgeR-robust)[34]. In order to account for subject-specific expression the quasi-likelihood F-test without any prior gene filtering was performed (edgeR-sensitive). Multiple testing correction was implemented using Benjamini-Hochberg procedure, significant DEGs were determined using false discovery rate (FDR) < 0.05 cutoff[35].

Gene Ontology (GO) terms and Kyoto Encyclopedia of Genes and Genomes (KEGG) pathways were adopted as the functional terms. GO and KEGG pathway enrichment analysis were performed with R package Goseq (1.30.0)[36]. KEGG pathways and GO terms with FDR < 0.05 were considered statistically significant.

Heat map was constructed with Matplotlib2 and SciPy3. Hierarchical clustering with average linkage method implemented in SciPy was used to cluster DEGs for contrasts M10h vs M0 and M7d vs M0 by their differences in read counts per million[37, 38].

Four-parameter algorithm in GraphPad Prism 8 was used for the calculation of sIgA concentrations in faecal samples and Wilcoxon signed rank test in R was applied for the evaluation of fluctuations in faecal sIgA levels among different time points. For the correlation analysis between sIgA levels and CPM values obtained in edgeR-robust analysis method, Spearman’s rank correlation test was performed in R, where significance level was set at 0.05.

## Results

### Differential global gene expression induced by administration of metformin

In order to reveal the target genes and pathways affected by metformin, we performed a transcriptome analysis in whole-blood samples of 25 healthy volunteers receiving metformin for one week (Table 1). Venous blood samples were obtained at three consecutive time-points, hereinafter referred to as M0 (before administration of metformin), M10h (10 hours after the first metformin intake/before the second dose) and M7d (after 7 days long metformin course) in order to observe both, acute and sustained effects of metformin. RNA-Sequencing produced an average of 24.6 ± 8.9 million reads per sample, 83.5% of the reads were mapped to the reference genome.

**Fig 2:**
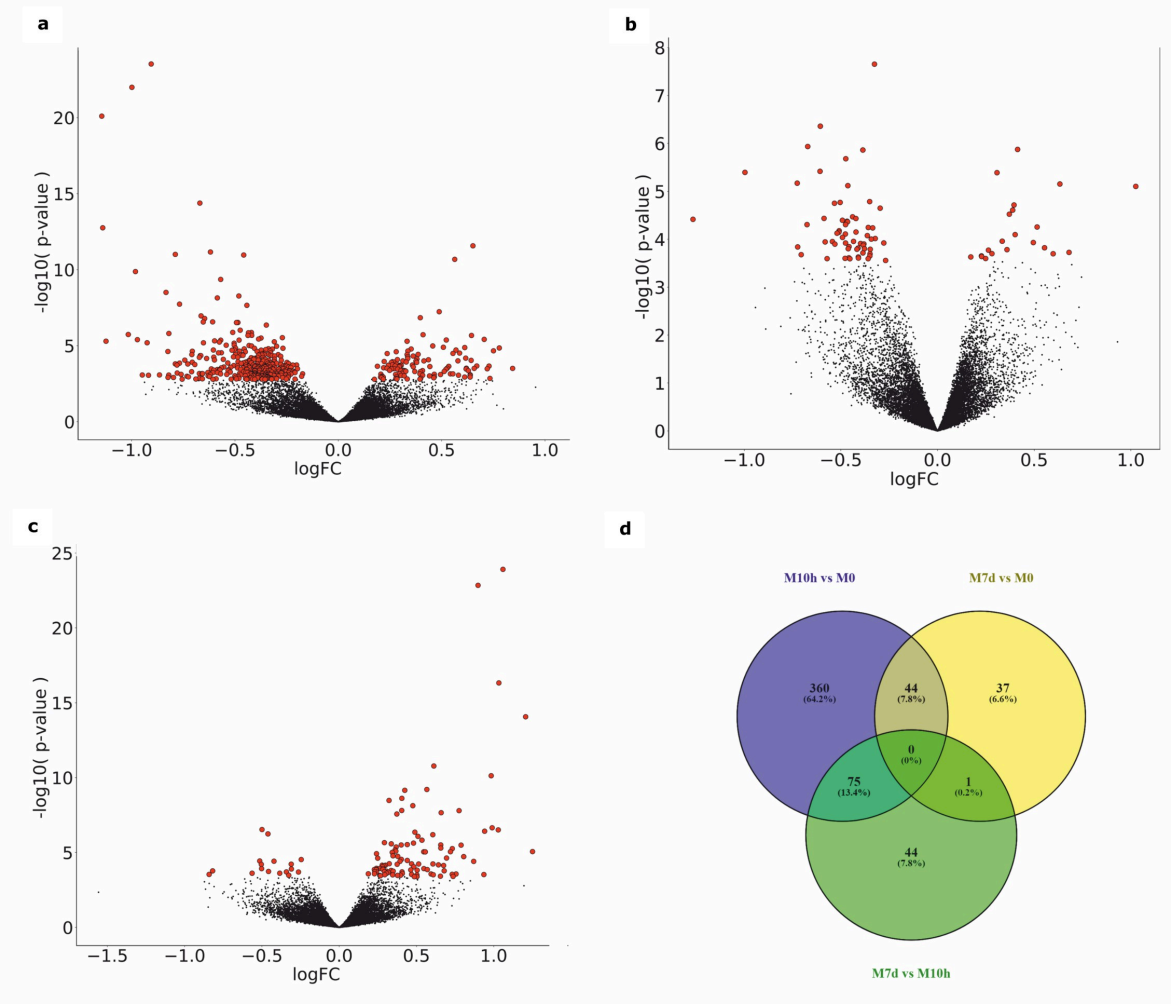
Metformin-induced alterations in gene expression profiles. Volcano plots showing the distribution of gene expression in the analyzed contrasts: (A)—M10h vs M0, (B)—M7d vs M0 and (C)—M7d vs M10h. Significance versus log_2_ fold change is plotted on the y and x axes, respectively. Red dots represent the significant DEGs (FDR < 0.05), black dots—nonsignificant genes. (D)—Venn diagram representing the number of total and overlapping significant DEGs in the analyzed contrasts obtained by edgeR-robust method.

**Fig 3:**
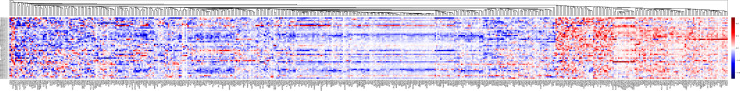
Heat map and hierarchical clustering of 517 DEGs in the contrasts M10h vs M0 and M7d vs M0. Each row corresponds to one subject in the respective contrast and each column represents a DEG. Normalized sequence read counts were rescaled to lie in range [0,1] and further used to estimate the difference between the gene expression levels in two time-points depending on the particular contrast. DEGs with analogous expression values were clustered at the column level, the list of DEGs were obtained by edgeR-robust method.

We focused on DEGs at three contrasts comparing the samples collected at previously defined time-points: M10h vs M0, M7d vs M0 and M7d vs M10h. In total 561 unique genes showed significantly different expression levels among the analyzed contrasts (Fig 2A, 2B, 2C and 2D). The majority, 479 of DEGs appeared in the contrast M10h vs M0 (364 downregulated and 115 upregulated). Comparison of M7d vs M0 resulted in 82 DEGs (61 downregulated, 21 upregulated) and 120 DEGs were identified in the contrast M7d vs M10h with higher proportion of upregulated genes (17 downregulated, 103 upregulated) (Fig 3). The overlap of the two main contrasts (M10h vs M0 and M7d vs M0) consisted of 44 DEGs, including *UBE2O*, *PHOSPHO1*, *MKRN1*, possessing consistent metformin-evoked alterations in expression levels for 7 days. The complete lists of obtained DEGs are provided in S1 Table.

### Functional annotation of the identified DEGs

In order to gain insights into the molecular mechanisms underlying the short-term effects of metformin the KEGG pathway enrichment analysis was performed. Three lists of DEGs corresponding to each contrast were submitted in Goseq package of R. DEGs obtained from the contrasts M10h vs M0 and M7d vs M10h were assigned to pathways related with immune responses, while list of DEGs obtained by comparing M7d samples with M0 samples showed no pathways enriched (Table 2). In order to gain a greater understanding of the biological implications of the obtained DEGs Gene Ontology (GO) enrichment analysis was performed, see S2 Table and S3 Table for the complete list of GO terms and KEGG pathways corresponding to DEGs obtained in all of the contrasts analyzed.

**Table 2:** Top KEGG pathways enriched by short-term metformin administration, ranked by statistical significance.

Contrast	Pathways	Count	Genes	Adjusted-P
**M10h vs M0**	Malaria	11	*GYPC*^*↓*^, *SELP*^*↓*^, *CD40LG*^*↑*^, *TLR2*^*↓*^, *CXCL8*^*↓*^, *HBA2*^*↓*^, *HBA1*^*↓*^, *CD40*^*↑*^, *THBS1*^*↓*^, *TGFB1*^*↓*^, ***SDC2***^*↓*^	7.88E-05
	Intestinal immune network for IgA production	10	*CXCR4*^*↓*^, *CD40LG*^*↑*^, *TNFSF13*^*↓*^, *CD40*^*↑*^, *HLA-DOA*^*↑*^, *MAP3K14*^*↑*^, ***HLA-DOB***^*↑*^, *TGFB1*^*↓*^, *HLA-DQA1*^*↑*^, *CD28*^*↑*^	7.88E-05
	Cytokine-cytokine receptor interaction	21	***TNFRSF21***^*↓*^, *IL1R2*^*↓*^, *FLT3*^*↓*^, *LEPR*^*↓*^, *CXCL8*^*↓*^, *TNFSF13*^*↓*^, *PF4*^*↓*^, *CD40*^*↑*^, *PF4V1*^*↓*^, *CCL4*^*↓*^, *TGFB1*^*↓*^, *ACVR1B*^*↓*^, *PPBP*^*↓*^, *CXCR4*^*↓*^, *CD40LG*^*↑*^, *EPOR*^*↓*^, *IL5RA*^*↓*^, *EGF*^*↓*^, *IL13RA1*^*↓*^, *IL3RA*^*↓*^, *CXCL5*^*↓*^.	7.88E-05
	Cell adhesion molecules (CAMs)	13	*ESAM*^*↓*^, ***CLDN5***^*↓*^, *HLA-DOB*^*↑*^, *SELP*^*↓*^, *HLA-DOA*^*↑*^, *CD28*^*↑*^, *PTPRF*^*↓*^, *CD8B*^*↑*^, *HLA-DQA1*^*↑*^, *CD40*^*↑*^, *VCAN*^*↓*^, *CD40LG*^*↑*^, *SDC2*^*↓*^.	6.49E-03
	Hematopoietic cell lineage	11	*MS4A1*^*↑*^, *IL5RA*^*↓*^, *IL3RA*^*↑*^, *CD8B*^*↑*^, ***FLT3***^*↓*^, *FCER2*^*↑*^, *CD19*^*↑*^, *GP9*^*↓*^, *IL1R2*^*↓*^, *ITGA2B*^*↓*^, *EPOR*^*↓*^.	6.49E-03
	Autoimmune thyroid disease	7	***HLA-DOB***^*↑*^, *HLA-DOA*^*↑*^, *CD28*^*↑*^, *GZMB*^*↑*^, *HLA-DQA1*^*↑*^, *CD40*^*↑*^, *CD40LG*^*↑*^.	6.49E-03
	Allograft rejection	7	***HLA-DOB***^*↑*^, *HLA-DOA*^*↑*^, *CD28*^*↑*^, *GZMB*^*↑*^, *HLA-DQA1*^*↑*^, *CD40*^*↑*^, *CD40LG*^*↑*^.	6.49E-03
	Rheumatoid arthritis	10	***HLA-DOB***^*↑*^, *HLA-DOA*^*↑*^, *CD28*^*↑*^, *TLR2*^*↓*^, *HLA-DQA1*^*↑*^, *CXCL5*^*↓*^, *TNFSF13*^*↓*^, *TGFB1*^*↓*^, *FOS*^*↓*^, *CXCL8*^*↓*^.	1.29E-02
	Graft-versus-host disease	7	*HLA-DOB*^*↑*^, *HLA-DOA*^*↑*^, *KLRD1*^*↓*^, *CD28*^*↑*^, *GZMB*^*↑*^, *HLA-DQA1*^*↑*^, ***KIR2DL1***^*↓*^.	1.49E-02
	Asthma	5	*HLA-DOB*^*↑*^, *HLA-DOA*^*↑*^, *HLA-DQA1*^*↑*^, *CD40*^*↑*^, *CD40LG*^*↑*^.	2.84E-02

Within each pathway one gene showing the highest expression variability, based on log_2_ fold change, is indicated in bold.

^↑^ Upregulated genes

^↓^ Downregulated genes

### Elevated faecal sIgA levels during the administration of metformin

**Fig 4:**
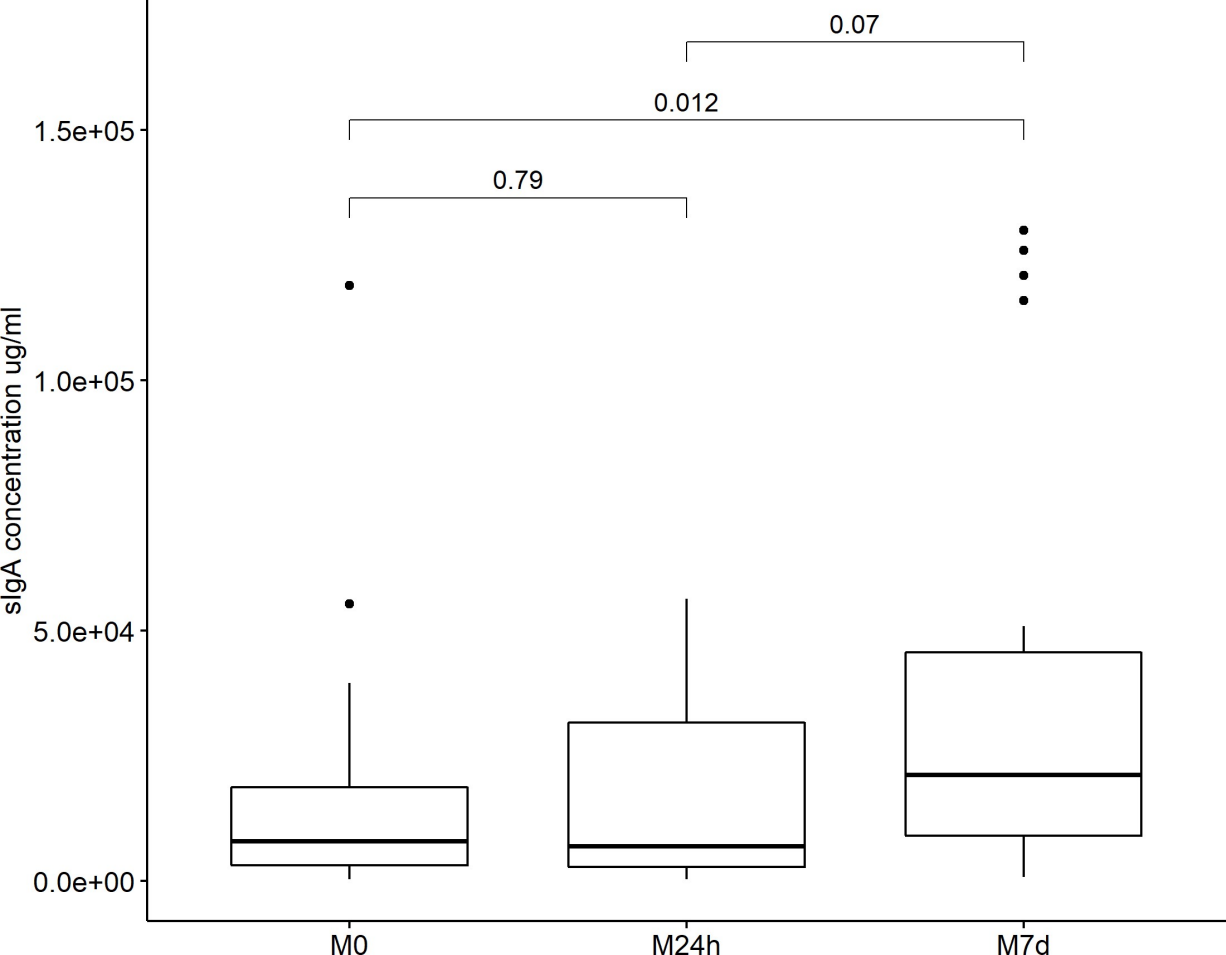
sIgA levels in stool samples during administration of metformin. Boxplot showing the difference in faecal sIgA levels at three time points: before administration of metformin (M0), 24 hours after the first dose (M24h) and 7 days after the first intake of metformin (M7d), measured by ELISA.

Considering the enrichment of pathways related to intestinal immune responses, sIgA concentration was determined in stool samples collected from the study participants at three consecutive time points, analogous to the course of blood sample collection: before administration of metformin (M0; median sIgA concentration = 7969.93μg/ml; IQR = 15587.55), within 24 hours after the first dose (M24h; median sIgA concentration = 6935.29μg/ml; IQR = 28953.46) and 7 days after the first intake of metformin (M7d; median sIgA concentration = 21207.64μg/ml; IQR = 36642.19), revealing significantly increased faecal sIgA levels comparing M7d samples vs M0 samples and M7d samples vs M24h samples (Fig 4).

The possible implication of specific immunity-related genes in intestinal IgA production in response to metformin administration was evaluated by Spearman’s rank correlation test between faecal sIgA concentration and expression level of DEGs involved in Intestinal immune network for IgA production and Cytokine-cytokine receptor interaction pathways, revealing 9 significant correlations (Table 3).

**Table 3:** Spearman’s correlation between faecal sIgA levels and expression of immunity-related genes.

Pathway	Gene	Spearman’s correlation coefficient	*p*-value
Intestinal immune network for IgA production	CXCR4*	0.40	5.15E-04
	***HLA-DQA1***	0.30	9.11E-03
	***MAP3K14***	0.25	3.01E-02
	*HLA-DOA*	0.19	1.17E-01
	*HLA-DOB*	0.16	1.64E-01
	*CD28*	-0.15	2.01E-01
	*TNFSF13* *	0.15	2.18E-01
	*CD40LG* *	0.07	5.30E-01
	*CD40* *	0.01	9.53E-01
	*TGFB1* *	0.14	2.42E-01
Cytokine-cytokine receptor interaction	***TNFRSF21***	0.32	6.54E-03
	***CCL4***	0.30	8.90E-03
	***ACVR1B***	0.26	2.44E-02
	***PF4***	0.25	3.29E-02
	***EPOR***	0.24	3.73E-02
	***CXCL8***	-0.24	3.92E-02
	*PF4V1*	0.21	7.07E-02
	*PPBP*	0.20	9.80E-02
	*FLT3*	0.12	2.96E-01
	*LEPR*	-0.09	4.49E-01
	*IL3RA*	0.08	4.77E-01
	*CXCL5*	-0.07	5.47E-01
	*EGF*	-0.02	8.59E-01
	*IL13RA1*	-0.01	9.57E-01
	*IL1R2*	0.01	9.63E-01
	*IL5RA*	0.00	9.75E-01

*Genes involved in both Intestinal immune network for IgA production and Cytokine-cytokine receptor interaction pathways.

Genes showing significant correlation with faecal sIgA levels are indicated in bold.

### Subject-specific effects of metformin

In order to clarify subject-specific metformin effects on the transcriptome profile, we performed an additional data analysis (quasi-likelihood F-test without any prior gene filtering hereinafter referred to as edgeR-sensitive), which allowed us to identify 437 unique DEGs among all of the contrasts (S1 Table; S1A, S1B, S1C and S1D Fig). After a careful inspection of the generated heat map for the contrasts M10h vs M0 and M7d vs M0, we observed striking inter-individual variation in expression levels of most significant DEGs (S2 Fig). It was evident that the overall differences in expression levels for a number of gene clusters were influenced by extreme changes in expression observed only in few individuals from the study. Four such functional clusters of genes with similar expression profiles were distinguished after consideration of subject-specific effects. In each of the recognized clusters, genes sharing a common function were predominant, including upregulated genes, coding for small nuclear RNAs (snRNAs): *SNORA20*, *SCARNA5*, *SNORA80E*, *SNORA3B*, *SCARNA22*, *SCARNA6*, *SNORD8*, *SNORA7B*, *SNORD9*, *SNORD83A*, *SNORA23*, *SNORD67*, *SNORD46*, *SNORA71B*, *SNORD71*, *SNORD11B*, *SNORD17*, *SNORD116-15*, *SNORD67*; ribosomal genes and pseudogenes together with long non-coding RNAs (lncRNAs): *Y_RNA*, *RNY4P10*, *RNA5-8SN1*, *RNA5-8SN5*, *FP671120*.*4*, *FP236383*.*2*, *FP671120*.*3*, *FP236383*.*3*, *RPL3P4*, *AC008038*.*1*, *AL122020*.*1*, *RPL13AP5*, *RPS2P46*, *RPL6P27*, *RPL13AP25*, *AL590867*, *RPS7P1*, *AP001324*.*1*, *AC034236*.*1*, *RPL37P2*, *AC004453*.*1*, *RPL21P16*, *AC007969*.*1*, *RPS23P8*, *RPL15P3*, *RPL10AP6*; genes relevant to insulin production: *HNF1B*, *HNF1A*, *HNF4A*, *GCK*, *INS*, *NEUROD1*, *PAX4*, *PDX1*, *ABCC8*, *KCNJ11* and downregulated genes involved in cholesterol homeostasis: *APOB*, *LDLR*, *PCSK9*.

## Discussion

This longitudinal study of metformin administration in healthy subjects demonstrated that metformin strongly affects the gene expression profiles in blood cells as estimated by RNA sequencing. To our knowledge this is the first study accessing the metformin-mediated changes in RNA expression *in vivo* in humans. Moreover, the significant transcriptomic changes were observed even after 10 hours as a result of single metformin dose, indicating the pronounced and immediate influence on the cell functions. The most striking finding was the strong evidence for metformin-induced enrichment of immunity-related pathways resulting in elevated faecal IgA levels.

Multiple DEGs identified in this study represent the main functional groups associated with previously described therapeutic targets of metformin. For instance, downregulation of gene coding for ubiquitin conjugating enzyme E2 O (*UBE2O*) impairs the tumorigenesis, moreover combined treatment of *UBE2O* inhibitors and AMPK agonists, such as metformin, has been suggested as promising treatment strategy for cancer already before[39]. Similarly, suppression of *MKRN1* (makorin ring finger protein 1) activates AMPK, resulting in increased glucose consumption and reduced lipid accumulation, therefore *MKRN1-*mediated regulation of AMPK activity has been already considered as an attractive therapeutic approach for the treatment of metabolic disorders[40]. Finally, DNA methylation at the *PHOSPHO1* (phosphoethanolamine/phosphocholine phosphatase 1) locus in blood cells has been previously linked to decreased type 2 diabetes mellitus risk, which goes in line with the well-known antidiabetic activity of metformin and metformin-evoked downregulation of *PHOSPHO1* observed in this study[41].

A group of discovered DEGs correspond to the pathways involved in immune response or regulation of inflammation. The pathway enrichment analysis showed comprehensively decreased expression levels of genes related to immune responses, which confirms the anti-inflammatory effect as a universal property of metformin. Here we convincingly support the previously described ability of metformin to suppress inflammatory cytokines and their receptors irrespective of the diabetes status, specifying the occurrence of this process at the level of mRNA[42]. The downregulation of *CXCL8* and *CXCR4*, coding for interleukin-8 and chemokine receptor type 4 respectively, has been previously attributed to the anticancer action of metformin in neoplastic cells, therefore the results of the present study describe the particular therapeutic effects as universal and characteristic also to the normal blood cells[43, 44].

Furthermore, RNA-Seq data revealed significant enrichment in the pathway of intestinal immune network for IgA production, which was further confirmed by elevated faecal sIgA in response to the metformin intervention. The observed correlation between faecal sIgA concentration and immunity-related gene expression levels points at transcriptional shift as a constitutive component of the intestinal immunity-related effects of metformin. IgA is responsible for bacteria-host interaction and is massively produced by mucosa in case of bacterial colonization; moreover, selective IgA deficiency is associated with mild intestinal dysbiosis and shifts in the microbial composition[45, 46]. Since the influence of metformin on gut microbiome is currently extensively studied[47], the potential implication of the intestinal immune network for IgA pathway in the metformin-microbiome interactions regarding the intestinal side effects should be considered.

In addition, we noticed subject-specific differences at gene expression levels, as number of individuals involved in study displayed overexpression or repression of specific functionally related gene sets. Thus for example insulin coding gene (*INS*) showed an apparent gain of the mRNA expression levels (from 0.52 CPM before the treatment to 282.97 CPM after one week of metformin administration) in a single individual. Based on the hierarchical clustering and functionality of these genes we categorized 4 gene clusters: ribosomal genes and their pseudogenes, small nuclear RNAs, genes relevant to insulin production and cholesterol homeostasis. Interestingly, the changes in RNA expression of those genes are highly subject-specific with strongly altered expression in only one or two participants of the study.

Metformin-induced overexpression of Maturity onset diabetes of the young (MODY)—related genes (*INS*, *PDX1*, *PAX4*, *HNF4A*, *HNF1A*, *HNF1B*, *NEUROD1*, *GCK*) coding for transcription factors and regulators of β-cell function was observed in both main comparisons (M10h vs M0, M7d vs M0), but not in the contrast M7d vs M10h, suggesting that these alterations are likely to be associated with metformin intervention rather than discrepancies between fasting and feasting states at the time points of blood collection. Metformin-induced differential expression of MODY genes has been previously reported in the liver of spontaneously hypertensive rats, overlapping several homologues of human genes[48]. Moreover, subject-specific activation of the MODY genes may be the reason why the metformin ability to induce insulin secretion has not been observed before in human trials. One may speculate that metformin exerts insulin secretagogue ability only in subgroup of metabolically compromised individuals, however, to prove this additional research in patients with metabolic syndrome and diabetes is needed.

Very similar to our detection of the MODY cluster our study revealed a considerable downregulation of the genes coding for apolipoprotein-B (APOB), low-density lipoprotein receptor (LDLR) and proprotein convertase subtilisin/kexin type 9 (PSCK9) in one person from the study group. All of these genes are previously associated with cholesterol homeostasis and phenotype of familial hypercholesterinaemia[49–51]. To date, several reports have described a dose-dependent cholesterol lowering effect of metformin[52, 53]. Taken together, it would be reasonable to argue that the downregulation of *LDLR*, *PCSK9*, *APOB* might serve as potential mechanism of action, underlying beneficial cardiovascular properties of metformin, yet in a case-specific manner.

The following are some study limitations. These limitations include small sample size and only one week of the intervention time due to the fact that study was performed on healthy individuals. Another limitation is that this is an exploratory study without a placebo control arm that would be needed to draw definitive conclusions on the causality of observed transcriptional changes. Also a larger study group of diabetic subjects and longer observation time would provide information on the factors that may explain subject-specific differences in expression levels and relation of these differences to the treatment response including glucose control.

These limitations are compensated by the longitudinal design of the study in which the first sample from an individual was the control for further samples. We believe that this design and short-term observation should have minimized false associations and conclusions arising from unaccounted factors playing important role in human studies, meanwhile making reasonable the interpretation of observed inter-individual variability of gene expression profiles.

In conclusion, we were able to provide, for the first time, direct evidence of the effects of metformin on the immediate and strong transcriptome changes in whole-blood samples. Our results have pinpointed some important targets that need further investigation. First, the ability of metformin to induce extensive immune responses may be executed at the level of transcription and serve as the basis of common therapeutic effect of metformin. Second, the induction of IgA pathway may explain the widely discussed interaction of metformin with the gut microbiome. Third, the subject-specific response may explain the large percent of unresponsiveness to the metformin therapy. Altogether these results may serve the ground for development of expression based biomarker sets to predict and/or monitor the treatment outcomes.

## Supporting information

S1 Table List of differentially expressed genes. (XLSX)

S2 Table Gene ontology analysis. (XLSX)

S3 Table KEGG pathway enrichment. (XLSX)

S4 Table Inclusion and exclusion criteria of the clinical trial ‘Pharmacodynamics of antidiabetic drug metformin’. (PDF)

S5 Table CONSORT Checklist. (PDF)

S1 Text Protocol of the clinical trial ‘Pharmacodynamics of antidiabetic drug metformin’. (PDF)

S1 Fig Metformin-induced alterations in gene expression profiles, considering subject-specific effects. Volcano plots showing the distribution of gene expression in the analyzed contrasts: (A)—M10h vs M0, (B)—M7d vs M0 and (C)—M7d vs M10h. Significance versus log2 fold change is plotted on the y and x axes, respectively. Red dots represent the significant DEGs (FDR < 0.05), black dots—nonsignificant genes. (D)—Venn diagram representing the number of total and overlapping significant DEGs in the analyzed contrasts, DEGs are obtained in the edgeR-sensitive analysis.
(TIFF)

S2 Fig Heat map and hierarchical clustering of 404 significant DEGs in the contrasts M10h vs M0 and M7d vs M0, accounting for subject-specific effects. Each row corresponds to one subject in the respective contrast and each column represents a DEG. Normalized sequence read counts were rescaled to lie in range [0,1] and further used to estimate the difference between the gene expression levels in two time-points depending on the particular contrast. DEGs with analogous expression values were clustered at the column level. Line plots show the expression levels (read counts per million) of the most representative genes of each subject-specific gene cluster in three blood sample collection time-points of one representative subject. DEGs are obtained in the edgeR-sensitive analysis.
(TIFF)

## References

[1] Standards of Medical Care in Diabetes-2017: Summary of Revisions. Diabetes Care. 2017;40(Suppl 1):S4–S5. doi: 10.2337/dc17-S003 27979887

[2] Johnson JA, Simpson SH, Toth EL, Majumdar SR. Reduced cardiovascular morbidity and mortality associated with metformin use in subjects with Type 2 diabetes. Diabet Med. 2005;22(4):497–502. doi: 10.1111/j.1464-5491.2005.01448.x 15787679

[3] Quinn BJ, Kitagawa H, Memmott RM, Gills JJ, Dennis PA. Repositioning metformin for cancer prevention and treatment. Trends Endocrinol Metab. 2013;24(9):469–80. doi: 10.1016/j.tem.2013.05.004 23773243

[4] Currie CJ, Poole CD, Jenkins-Jones S, Gale EA, Johnson JA, Morgan CL. Mortality after incident cancer in people with and without type 2 diabetes: impact of metformin on survival. Diabetes Care. 2012;35(2):299–304. doi: 10.2337/dc11-1313 22266734 PMC3263862

[5] Velazquez EM, Mendoza S, Hamer T, Sosa F, Glueck CJ. Metformin therapy in polycystic ovary syndrome reduces hyperinsulinemia, insulin resistance, hyperandrogenemia, and systolic blood pressure, while facilitating normal menses and pregnancy. Metabolism. 1994;43(5):647–54. doi: 10.1016/0026-0495(94)90209-7 8177055

[6] Tang T, Lord JM, Norman RJ, Yasmin E, Balen AH. Insulin-sensitising drugs (metformin, rosiglitazone, pioglitazone, D-chiro-inositol) for women with polycystic ovary syndrome, oligo amenorrhoea and subfertility. Cochrane Database Syst Rev. 2012(5):CD003053. doi: 10.1002/14651858.CD003053.pub5 22592687

[7] Gupta A, Bisht B, Dey CS. Peripheral insulin-sensitizer drug metformin ameliorates neuronal insulin resistance and Alzheimer's-like changes. Neuropharmacology. 2011;60(6):910–20. doi: 10.1016/j.neuropharm.2011.01.033 21277873

[8] Asadbegi M, Yaghmaei P, Salehi I, Ebrahim-Habibi A, Komaki A. Neuroprotective effects of metformin against Abeta-mediated inhibition of long-term potentiation in rats fed a high-fat diet. Brain Res Bull. 2016;121:178–85. doi: 10.1016/j.brainresbull.2016.02.005 26861514

[9] Mendelsohn AR, Larrick JW. Rapamycin as an antiaging therapeutic?: targeting mammalian target of rapamycin to treat Hutchinson-Gilford progeria and neurodegenerative diseases. Rejuvenation Res. 2011;14(4):437–41. doi: 10.1089/rej.2011.1238 21851176

[10] Zhou G, Myers R, Li Y, Chen Y, Shen X, Fenyk-Melody J, et al. Role of AMP-activated protein kinase in mechanism of metformin action. J Clin Invest. 2001;108(8):1167–74. doi: 10.1172/JCI13505 11602624 PMC209533

[11] Miller RA, Chu Q, Xie J, Foretz M, Viollet B, Birnbaum MJ. Biguanides suppress hepatic glucagon signalling by decreasing production of cyclic AMP. Nature. 2013;494(7436):256–60. doi: 10.1038/nature11808 23292513 PMC3573218

[12] El-Mir MY, Nogueira V, Fontaine E, Averet N, Rigoulet M, Leverve X. Dimethylbiguanide inhibits cell respiration via an indirect effect targeted on the respiratory chain complex I. J Biol Chem. 2000;275(1):223–8. doi: 10.1074/jbc.275.1.223 10617608

[13] Wu H, Esteve E, Tremaroli V, Khan MT, Caesar R, Manneras-Holm L, et al. Metformin alters the gut microbiome of individuals with treatment-naive type 2 diabetes, contributing to the therapeutic effects of the drug. Nat Med. 2017;23(7):850–8. doi: 10.1038/nm.4345 28530702

[14] Cook MN, Girman CJ, Stein PP, Alexander CM. Initial monotherapy with either metformin or sulphonylureas often fails to achieve or maintain current glycaemic goals in patients with Type 2 diabetes in UK primary care. Diabet Med. 2007;24(4):350–8. doi: 10.1111/j.1464-5491.2007.02078.x 17335466

[15] Kahn SE, Haffner SM, Heise MA, Herman WH, Holman RR, Jones NP, et al. Glycemic durability of rosiglitazone, metformin, or glyburide monotherapy. N Engl J Med. 2006;355(23):2427–43. doi: 10.1056/NEJMoa066224 17145742

[16] Garber AJ, Duncan TG, Goodman AM, Mills DJ, Rohlf JL. Efficacy of metformin in type II diabetes: results of a double-blind, placebo-controlled, dose-response trial. Am J Med. 1997;103(6):491–7. doi: 10.1016/s0002-9343(97)00254-4 9428832

[17] Zhou K, Donnelly L, Yang J, Li M, Deshmukh H, Van Zuydam N, et al. Heritability of variation in glycaemic response to metformin: a genome-wide complex trait analysis. Lancet Diabetes Endocrinol. 2014;2(6):481–7. doi: 10.1016/S2213-8587(14)70050-6 24731673 PMC4038749

[18] Tkac I, Klimcakova L, Javorsky M, Fabianova M, Schroner Z, Hermanova H, et al. Pharmacogenomic association between a variant in SLC47A1 gene and therapeutic response to metformin in type 2 diabetes. Diabetes Obes Metab. 2013;15(2):189–91. doi: 10.1111/j.1463-1326.2012.01691.x 22882994

[19] Shikata E, Yamamoto R, Takane H, Shigemasa C, Ikeda T, Otsubo K, et al. Human organic cation transporter (OCT1 and OCT2) gene polymorphisms and therapeutic effects of metformin. J Hum Genet. 2007;52(2):117–22. doi: 10.1007/s10038-006-0087-0 17111267

[20] Choi JH, Yee SW, Ramirez AH, Morrissey KM, Jang GH, Joski PJ, et al. A common 5'-UTR variant in MATE2-K is associated with poor response to metformin. Clin Pharmacol Ther. 2011;90(5):674–84. doi: 10.1038/clpt.2011.165 21956618 PMC3329222

[21] Chen L, Pawlikowski B, Schlessinger A, More SS, Stryke D, Johns SJ, et al. Role of organic cation transporter 3 (SLC22A3) and its missense variants in the pharmacologic action of metformin. Pharmacogenet Genomics. 2010;20(11):687–99. doi: 10.1097/FPC.0b013e32833fe789 20859243 PMC2976715

[22] Jablonski KA, McAteer JB, de Bakker PI, Franks PW, Pollin TI, Hanson RL, et al. Common variants in 40 genes assessed for diabetes incidence and response to metformin and lifestyle intervention in the diabetes prevention program. Diabetes. 2010;59(10):2672–81. doi: 10.2337/db10-0543 20682687 PMC3279522

[23] Rotroff DM, Yee SW, Zhou K, Marvel SW, Shah HS, Jack JR, et al. Genetic Variants in CPA6 and PRPF31 Are Associated With Variation in Response to Metformin in Individuals With Type 2 Diabetes. Diabetes. 2018;67(7):1428–40. doi: 10.2337/db17-1164 29650774 PMC6014560

[24] GoDarts, Group UDPS, Wellcome Trust Case Control C, Zhou K, Bellenguez C, Spencer CC, et al. Common variants near ATM are associated with glycemic response to metformin in type 2 diabetes. Nat Genet. 2011;43(2):117–20. doi: 10.1038/ng.735 21186350 PMC3030919

[25] Niu N, Liu T, Cairns J, Ly RC, Tan X, Deng M, et al. Metformin pharmacogenomics: a genome-wide association study to identify genetic and epigenetic biomarkers involved in metformin anticancer response using human lymphoblastoid cell lines. Hum Mol Genet. 2016;25(21):4819–34. doi: 10.1093/hmg/ddw301 28173075 PMC6078562

[26] Pawlyk AC, Giacomini KM, McKeon C, Shuldiner AR, Florez JC. Metformin pharmacogenomics: current status and future directions. Diabetes. 2014;63(8):2590–9. doi: 10.2337/db13-1367 25060887 PMC4113063

[27] Guo J, Zhou Y, Cheng Y, Fang W, Hu G, Wei J, et al. Metformin-Induced Changes of the Coding Transcriptome and Non-Coding RNAs in the Livers of Non-Alcoholic Fatty Liver Disease Mice. Cell Physiol Biochem. 2018;45(4):1487–505. doi: 10.1159/000487575 29466788

[28] Martin-Montalvo A, Mercken EM, Mitchell SJ, Palacios HH, Mote PL, Scheibye-Knudsen M, et al. Metformin improves healthspan and lifespan in mice. Nat Commun. 2013;4:2192. doi: 10.1038/ncomms3192 23900241 PMC3736576

[29] Padilla J, Thorne PK, Martin JS, Rector RS, Akter S, Davis JW, et al. Transcriptomic effects of metformin in skeletal muscle arteries of obese insulin-resistant rats. Exp Biol Med (Maywood). 2017;242(6):617–24. 28114814 10.1177/1535370216689825 PMC5685263

[30] Udhane SS, Legeza B, Marti N, Hertig D, Diserens G, Nuoffer JM, et al. Combined transcriptome and metabolome analyses of metformin effects reveal novel links between metabolic networks in steroidogenic systems. Sci Rep. 2017;7(1):8652. doi: 10.1038/s41598-017-09189-y 28819133 PMC5561186

[31] Sacco F, Silvestri A, Posca D, Pirro S, Gherardini PF, Castagnoli L, et al. Deep Proteomics of Breast Cancer Cells Reveals that Metformin Rewires Signaling Networks Away from a Pro-growth State. Cell Syst. 2016;2(3):159–71. doi: 10.1016/j.cels.2016.02.005 27135362

[32] Rovite V, Wolff-Sagi Y, Zaharenko L, Nikitina-Zake L, Grens E, Klovins J. Genome Database of the Latvian Population (LGDB): Design, Goals, and Primary Results. J Epidemiol. 2018. 10.2188/jea.JE20170079 PMC6048300 29576601

[33] Zhang M, Liu YH, Chang CS, Zhi H, Wang S, Xu W, et al. Quantification of gene expression while taking into account RNA alternative splicing. Genomics. 2018. 10.1016/j.ygeno.2018.10.009 30366041

[34] Zhou X, Lindsay H, Robinson MD. Robustly detecting differential expression in RNA sequencing data using observation weights. Nucleic Acids Res. 2014;42(11):e91. doi: 10.1093/nar/gku310 24753412 PMC4066750

[35] Benjamini YaH, Y. Controlling the False Discovery Rate: A Practical and Powerful Approach to Multiple Testing. Journal of the Royal Statistical Society, Series B (Methodological). 1995;57(1).

[36] Young MD, Wakefield MJ, Smyth GK, Oshlack A. Gene ontology analysis for RNA-seq: accounting for selection bias. Genome Biol. 2010;11(2):R14. doi: 10.1186/gb-2010-11-2-r14 20132535 PMC2872874

[37] P JEOTP. SciPy: Open Source Scientific Tools for Python. Computing in Science & Engineering. 2007;9:10–20.

[38] Hunter JD. Matplotlib: A 2D graphics environment. Computing in Science & Engineering. 2007;9:99–104.

[39] Vila IK, Song SJ, Song MS. A new duet in cancer biology: AMPK the typical and UBE2O the atypical. Mol Cell Oncol. 2017;4(3):e1304846. doi: 10.1080/23723556.2017.1304846 28616582 PMC5462509

[40] Lee MS, Han HJ, Han SY, Kim IY, Chae S, Lee CS, et al. Loss of the E3 ubiquitin ligase MKRN1 represses diet-induced metabolic syndrome through AMPK activation. Nat Commun. 2018;9(1):3404. doi: 10.1038/s41467-018-05721-4 30143610 PMC6109074

[41] Dayeh T, Tuomi T, Almgren P, Perfilyev A, Jansson PA, de Mello VD, et al. DNA methylation of loci within ABCG1 and PHOSPHO1 in blood DNA is associated with future type 2 diabetes risk. Epigenetics. 2016;11(7):482–8. doi: 10.1080/15592294.2016.1178418 27148772 PMC4939923

[42] Cameron AR, Morrison VL, Levin D, Mohan M, Forteath C, Beall C, et al. Anti-Inflammatory Effects of Metformin Irrespective of Diabetes Status. Circ Res. 2016;119(5):652–65. doi: 10.1161/CIRCRESAHA.116.308445 27418629 PMC4990459

[43] Bruno S, Ledda B, Tenca C, Ravera S, Orengo AM, Mazzarello AN, et al. Metformin inhibits cell cycle progression of B-cell chronic lymphocytic leukemia cells. Oncotarget. 2015;6(26):22624–40. doi: 10.18632/oncotarget.4168 26265439 PMC4673187

[44] Xiao Z, Wu W, Poltoratsky V. Metformin Suppressed CXCL8 Expression and Cell Migration in HEK293/TLR4 Cell Line. Mediators Inflamm. 2017;2017:6589423. doi: 10.1155/2017/6589423 29147073 PMC5632916

[45] Gutzeit C, Magri G, Cerutti A. Intestinal IgA production and its role in host-microbe interaction. Immunol Rev. 2014;260(1):76–85. doi: 10.1111/imr.12189 24942683 PMC4174397

[46] Fadlallah J, El Kafsi H, Sterlin D, Juste C, Parizot C, Dorgham K, et al. Microbial ecology perturbation in human IgA deficiency. Sci Transl Med. 2018;10(439). 10.1126/scitranslmed.aan1217 29720448

[47] Elbere I, Kalnina I, Silamikelis I, Konrade I, Zaharenko L, Sekace K, et al. Association of metformin administration with gut microbiome dysbiosis in healthy volunteers. PLoS One. 2018;13(9):e0204317. doi: 10.1371/journal.pone.0204317 30261008 PMC6160085

[48] Malinska H, Oliyarnyk O, Skop V, Silhavy J, Landa V, Zidek V, et al. Effects of Metformin on Tissue Oxidative and Dicarbonyl Stress in Transgenic Spontaneously Hypertensive Rats Expressing Human C-Reactive Protein. PLoS One. 2016;11(3):e0150924. doi: 10.1371/journal.pone.0150924 26963617 PMC4786274

[49] Radovica-Spalvina I, Latkovskis G, Silamikelis I, Fridmanis D, Elbere I, Ventins K, et al. Next-generation-sequencing-based identification of familial hypercholesterolemia-related mutations in subjects with increased LDL-C levels in a latvian population. BMC Med Genet. 2015;16:86. doi: 10.1186/s12881-015-0230-x 26415676 PMC4587402

[50] Dixon JL, Ginsberg HN. Regulation of hepatic secretion of apolipoprotein B-containing lipoproteins: information obtained from cultured liver cells. J Lipid Res. 1993;34(2):167–79. 8381452

[51] Trapani L, Segatto M, Pallottini V. Regulation and deregulation of cholesterol homeostasis: The liver as a metabolic "power station". World J Hepatol. 2012;4(6):184–90. doi: 10.4254/wjh.v4.i6.184 22761969 PMC3388116

[52] Wulffele MG, Kooy A, de Zeeuw D, Stehouwer CD, Gansevoort RT. The effect of metformin on blood pressure, plasma cholesterol and triglycerides in type 2 diabetes mellitus: a systematic review. J Intern Med. 2004;256(1):1–14. doi: 10.1111/j.1365-2796.2004.01328.x 15189360

[53] Pentikainen PJ, Voutilainen E, Aro A, Uusitupa M, Penttila I, Vapaatalo H. Cholesterol lowering effect of metformin in combined hyperlipidemia: placebo controlled double blind trial. Ann Med. 1990;22(5):307–12. doi: 10.3109/07853899009147912 2291838

