# Supplementary material for: Metformin strongly affects transcriptome of peripheral blood cells in healthy individuals
Source: PLoS One. 2019 Nov 8;14(11):e0224835. doi: 10.1371/journal.pone.0224835 (PMC6839856; doi:10.1371/journal.pone.0224835)
Supplement: S4 Table — (PDF) [file pone.0224835.s004.pdf]

Metformin Strongly Affects Transcriptome of Peripheral Blood Cells in Healthy Individuals

Monta Ustinova, Ivars Silamikelis, Ineta Kalnina, Laura Ansone, Vita Rovite, Ilze Elbere, Ilze Radovica-Spalvina, Davids Fridmanis, Jekaterina Aladyeva, Ilze Konrade, Valdis Pirags, Janis Klovins.

S4 Table. Inclusion and exclusion criteria of the clinical trial 'Pharmacodynamics of antidiabetic drug metformin'.

**Principal inclusion criteria**
1. Healthy person with no known illnesses at the time of application that could possibly alter the results of the study;
2. Body characteristic parameters (e.g. weight) are within the conventional range;
3. Mental condition allows a person to understand the research process and give a legal consent for the participation in it;
4. Age: 18 – 64 years;
5. European descent;
6. Both women and men with reproductive potential correspond to the contraceptive requirements stated in the study protocol;
7. Prior to the study-related procedures, the consent of a person's participation in the clinical trial is received by submitting a signed and dated informed consent document.
**Principal exclusion criteria**
1. Allergies to any of *Metforal* 850mg components;
2. Usage of any other medication which is not compatible with *Metforal* 850mg;
3. Pregnancy or lactation;
4. Type 1 or type 2 diabetes, pancreatogenic diabetes, impaired glucose tolerance;
5. Polycystic ovarian syndrome;
6. Chronic diseases of intestinal tract, oncological or autoimmune diseases;
7. Renal failure or renal impairment;
8. Hepatic impairment or alcoholism;
9. Acute conditions with the potential effects on kidney;
10. Acute or chronic disease which may cause tissue hypoxia;
11. Diarrhea during the past week;
12. Long term previous administration of metformin;
13. Use of the following products during the previous two months:<br>a. antibiotics,<br>b. probiotics,<br>c. proton pump inhibitors,<br>d. immunosuppressive agents,<br>e. corticosteroids;

14. Intravascular administration of iodinated contrast agents intended during the active period of the clinical trial.

ALAT - alanine aminotransferase; HbA1c - hemoglobin A1c

Inclusion and exclusion criteria of the clinical trial.
