# Supplementary material for: Metformin strongly affects transcriptome of peripheral blood cells in healthy individuals
Source: PLoS One. 2019 Nov 8;14(11):e0224835. doi: 10.1371/journal.pone.0224835 (PMC6839856; doi:10.1371/journal.pone.0224835)
Supplement: S1 Text — (PDF) [file pone.0224835.s006.pdf]

PHARMACODYNAMIC STUDY OF ANTIDIABETIC DRUG METFORMIN
Research Protocol

Part1

PROJECT SUMMARY

Metformin is a widely used first-line antidiabetic agent for treatment of type 2 diabetes mellitus (T2D). Despite its advantages, metformin has variable therapeutic effects, contraindications, and side effects. There is growing evidence regarding contribution of the epigenetic mechanisms in reaching metformin's therapeutic goals, however, the effect of metformin on human cells *in vivo* is not comprehensively studied. In addition, a possible relevance of alterations in the composition of gut microbiota has been found in metformin-related gastrointestinal side effects. Here, for the very first time, we investigate the short-term effect of metformin on the composition of healthy human gut microbiota, DNA methylation and transcriptome profiles.

The aim is to perform a wide-spectrum study exploring mechanisms of action of antidiabetic agent metformin by performing analysis on blood cell derived genetic material and gut microbiome samples.

We will perform an exploratory longitudinal study design in which the first sample from an individual will serve as the control for further samples. Fifty healthy individuals will be treated with *Metforal* 850 mg (2 × 850 mg of Metformin Hydrochloride) for 7 days. Stool samples will be collected at three time points: prior to administration, 24 hours and 7 days after metformin administration in order to determine the taxonomic composition of the gut microbiome by massive parallel sequencing. For DNA methylation and transcriptome analysis in white blood cells, blood samples will be collected before the administration of metformin, 10h after the first dose and after 7-day period of metformin therapy.

The results of the trial will provide a comprehensive information of the metformin-induced alterations in the composition of gut microbiome, DNA methylation and transcriptome profiles, revealing the underlying molecular mechanisms of metformin action. This approach may deliver new knowledge for development of personalized approaches in T2D treatment especially to overcome issues of inter-individual variability in antidiabetic therapy.

GENERAL INFORMATION

Protocol title: Pharmacodynamics of antidiabetic drug metformin
EudraCT number: 2016-001092-74
Sponsor's protocol code number: MIKROMET16001
Sponsor's protocol version: 3
Sponsor's protocol date: 2017-03-14

Sponsor

Name of organization: Latvian Biomedical Research and Study centre
Name of the person to contact: Janis Klovins
Address: Ratsupites Str. 1 k-1, Riga, Latvia, LV-1067.
E-mail: BMC@biomed.lu.lv

Investigator

Name of the principal investigator: Valdis Pirags
Qualification: Prof., Dr.med. Endocrinologist
Institution name: Latvian Biomedical Research and Study Centre
Address: Ratsupites Str. 1 k-1, Riga, Latvia, LV-1067.
E-mail: pirags@latnet.lv

Research site

Name of organization: Latvian Biomedical Research and Study centre
Address: Ratsupites Str. 1 k-1, Riga, Latvia, LV-1067.
E-mail: BMC@biomed.lu.lv

Other medical or technical institutions involved

Name of organization: Latvian Biomedical Research and Study centre
Department: Genome Centre, Biobank unit
Address: Ratsupites Str. 1 k-1, Riga, Latvia, LV-1067
E-mail: gec@biomed.lu.lv

Name of organization: E. Gulbis Laboratory
Address: 366 Brivibas gatve, Riga, Latvia, LV-1006
E-mail: Ilze.lindenberga@egl.lv

Name of organization: Uppsala University
Department: Uppsala Biomedical Centre
Address Husargatan 3, Uppsala, Sweden, 751 24
E-mail: Helgi.Schioth@neuro.uu.se

Name of organization: Latvian Institute of Organic Synthesis
Address: 21 Aizkraukles street, Riga, Latvia, LV-1006
E-mail: osvalds@osi.lv

RATIONALE & BACKGROUND INFORMATION

According to the World Health Organization, diabetes mellitus is affecting approximately 60 million people in the European Region (>80000 people in Latvia are receiving diabetes treatment), and is associated with premature death and increased incidence of complications. Metformin is the most commonly used first line antidiabetic drug in treatment of T2D and has been widely prescribed to treat other diseases. Metformin as insulin sensitizer drug exerts its activity by inhibiting gluconeogenesis in the liver and subsequently lowering glucose levels in the blood [Jakab 2018].

Two main issues concerning the metformin use can be put forward: (1) unfortunately up to 30% of patients using metformin monotherapy do not reach glycaemic goals and have to switch to other therapies, and (2) 10-20% of metformin users experience gastrointestinal side-effects leading to the significant non-compliance and discontinuation of therapy. Given the scale to which the metformin is used worldwide (13th most prescribed drug in USA), above mentioned numbers have enormous impact on the significant proportion of population.

In recent study metformin was shown to modulate gut microbiota composition, for example by increasing some bacteria species (e.g. *Akkermansia muciniphila*) [de la Cuesta-Zuluaga et al., 2017]. In T2D research large metagenome-wide studies have described gut microbial dysbiosis in obese and T2D patients, including a reduction in butyrate-producing bacteria and an increase in opportunistic pathogens. Experiments in rodents suggest that metformin may exert its effects via the modified microbiome [Shin et al., 2014]. Impact of gut microbiota on side effects of metformin action in humans has not been investigated so far in a prospective study. We, therefore, will estimate the metformin introduced changes in the gut microbiota in healthy people comparing the 16S rRNA gene sequencing and shotgun metagenomic analysis results of fecal samples before and after metformin use.

As far as pharmacogenetics of metformin is concerned, previous studies have extensively investigated genetic variation in metformin transporters (OCT1, OCT2, OCT3, MATE1, MATE2, PMAT) with limited success, and only one GWAS have been published so far. We were the first to publish that genetic variation in OCT1 gene is associated with metformin side effects [Tarasova et al., 2012] that has been partially supported by other study [Dujic et al., 2016]. Lately it has been shown that epigenetics may play a key role in the pathogenesis of T2D [Nilsson et al., 2014] including the finding that blood DNA methylation is altered at some genes which was suggested to reflect T2D progression from twins discordant for T2D [Yuan et al., 2014] as well as DNA hypomethylation of specific sites in young individuals who later developed T2D [Toperoff et al. 2012]. Metformin's effects on DNA methylation as well as associated transcriptome changes still remain to be investigated. We therefore will perform genome wide DNA methylation (Illumina BeadChip based) and transcriptome analysis (RNA-Seq) in healthy humans after metformin intake at several time points. The identification of epigenetic biomarkers, combined with genetic and environmental factors, will optimize the therapeutics of T2D achieving the treatment and

prediction of these pathologies at an early stage, accelerating the discovery of new therapies, lowering the economic and social costs and introducing high quality innovation to this field.

Genetic variation and epigenetic importance in effectiveness of metformin therapy

Pharmacogenetic studies performed so far have identified changes in receptor proteins, transport proteins and metabolic enzyme encoding gene sequences, which explains effectiveness and toxicity of variable drugs among T2D patients [Gong et al., 2012]. Allelic variants have been identified in genes SLC22A1, SLC22A2, SLC22A3, SLC47A1 and SLC47A1 with functional relevance to the pharmacokinetic and pharmacodynamics of metformin, but no biomarker has yet been validated allowing to predict efficiency of metformin and the response of the body to metformin during therapy [Becker et al., 2010, He et al., 2015, Gong et al. 2012, Song et al.,2008].

In addition of changes in functional gene sequences, heterogeneous DNA methylation in drug-related metabolic locus is one of the potential causes of variable therapeutic efficacy among T2D patients. Studies have shown that epigenetic changes may play an important role in the pathogenesis of T2D [Nilsson et al., 2014]. In twins that differ in T2D status the methylation profile has been changed in specific genes associated with the development of the disease [Yuan et al., 2014]. Among young people, hypermethylation in certain genome regions has been determined to be associated with the development of T2D in later years [Toperoff et al., 2012]. The DNA methylation profile appears to differ in the main tissues associated with the maintenance of glucose homeostasis, pancreatic beta cells, liver and skeletal muscles, between T2D patients and individuals with normal glucose tolerance (Dayeh et al., 2014). Diet and level of physical activities undeniably affect clinical signs of T2D. Both of these factors are associated with changes in the level of methylation in peripheral tissues, including those gene loci whose functions are related to the regulation of insulin secretion and insulin signaling pathways [Barres et al., 2012; Jacobsen et al., 2012; Ronn and Ling, 2015]. Thus, the given epigenetic mechanism provides a link between environmental factors and regulation of genes involved in metabolism [Martinez et al., 2014; Ronn and Ling, 2015]. Contrary of original beliefs, it is dynamic and potentially reversible process. For example, in animal model with high-fat diet followed by standard diet, the proposed changes in DNA methylation was restored recovering the previous profile [Uriarte et al., 2013; Martinez et al., 2014]. Similarly, in patients with obese skeletal muscle, changes in methylation were eliminated in genes that encode mitochondrial functions regulatory proteins in the promoter regions. After weight reduction by gastric bypass surgery, the level of methylation returned to the characteristic of controls [Barres et al., 2013; Martinez et al., 2014]. The potential reversible process of the DNA methylation,

makes epigenetic changes a promising target for the development of new therapies and the development of biomarkers for monitoring existing drug therapies.

Changes in DNA methylation profiles have already been used as a predicted marker for evaluating the effectiveness of chemotherapy in patients with different malignant tumors [Coppedè et al. 2014, Mikeska and Craig 2014]. In addition, the association of metformin with changes in DNA methylation profiles in breast and endometrial malignant cells is described, but not in T2D patients or healthy subjects. Individual methylated loci are statistically associated with metformin cytotoxicity (IC50) [Niu et al. 2016, Zhong et al. 2016]. The effect of metformin on changes in the DNA methylation profile and therefore in transcriptome in the context of diabetes therapy have not been studied until now. In theory, metformin could influence the methylation profile to regulate metabolism and susceptibility to insulin. By establishing the DNA methylation profile and gene regulation in white cells, it would be possible to gain an idea of the molecular processes in targeted organs that are otherwise difficult to access due to complicated sampling. Supplementing the intestinal microbiome analysis profile with DNA methylation data, new information on the effect of intestinal microflora to the metabolism of the host at the level of gene regulation could be obtained in the context of metformin therapy. Similarly, it has been shown that gut microflora, by metabolizing different components, modulates the effect of diet to the DNA methylation profile of enterocytes accordingly. In view of the fact, that many of microflora metabolites enter circulation, gene regulation in other organs is likely to be affected [Hullar and Fu, 2014]. On the clinical side, clarifying the DNA methylation profiles associated with metformin usage, in the future would allow to develop an epigenetic marker panel for predicting the outcome of therapy in relatively non-invasive process from blood derived samples.

The gut microbiome and therapeutic efficacy of metformin

Therapeutic effect of metformin is variable, as well as observed contraindications and side effects, which means that a personalized approach is necessary to apply a medication therapy strategy [Nasri and Rafieian-Kopaei 2014]. Metformin action in the gastrointestinal tract has been acknowledged to have a significant role in the achievement of its therapeutic effect and, possibly, in the development of associated side effects. The concentration of metformin in the small intestine is about 30-300 times higher than plasma concentrations, and approximately 30% of the drug is eliminated with feces [Graham et al., 2011; McCreight et al., 2016]. Also, delayed-release metformin that is absorbed in distal small intestine, thus with lower bioavailability (up to 50%), has shown equal or higher glucose lowering effect compared

to immediate-release metformin [Buse et al., 2016; et al., 2016]. The drug effect in the gut is associated with altered glucose absorption and increased glucose metabolism. As result, enterocytes increase lactate production, that could partly explain gastrointestinal side effects, decreased reabsorption of bile acids from the intestines that cause changes in gut microbiome composition and increase secretion of glucagon-like-peptide-1 (GLP-1) [Scarpello et al., 1998; Carter et al., 2003; Buse et al., 2016; McCreight et al., 2016; Wu et al., 2016].

Lately, more attention is devoted to understanding the role of the gut microbiome in the therapeutic effect of metformin and caused side effects. Studies in animals have shown that metformin increase the abundance of *Akkermansia spp*. that are mucin degrading bacteria [Shin et al., 2014]. After increasing *Akkermansia muciniphila* proportion in mouse microbiome, improved glucose tolerance was observed, the effect resulted from increased insulin sensitivity in the liver and in the muscle tissue. These alterations in metabolism were associated with inhibited gluconeogenesis, reduced fat accumulation in the liver, alleviated endoplasmic reticulum (ER) stress in liver and muscle and with reduced inflammation status. Most likely positive effect is achieved by *Akkermansia spp*. mediated intestinal mucosa regeneration, that results in a reduced amount of inflammatory lipopolysaccharides (LPS) entering the host organism [Zhao et al., 2016].

Studies in human populations support the hypothesis of metformin interaction with gut microbiome. For example, T2D patients that use metformin have a higher proportion of short-chain fatty acid producing bacteria like *Subdoligranulum spp*., *Akkermansia spp*., *Bifidobacterium bifidum*, when compared with patients that use different therapy [Forslund et al., 2015; Zhang et al., 2015; de la Cuesta-Zuluaga et al., 2017]. Short-chain fatty acids, like butyrate, propionate, and acetate, have a beneficial effect on glucose tolerance and overall energy homeostasis in the organism [De Vadder et al., 2014]. As mentioned before, *Akkermansia spp*. improves the state of intestinal mucosa therefore ensuring an additional effect on stabilization of metabolism [Forslund et al., 2015; Zhao et al., 2016; de la Cuesta-Zuluaga et al., 2017]. These observations show that at least part of the metformin glucose lowering effect could be explained with altered microbiome composition. In addition, an increased amount of bacteria from *Escherichia* genus was observed in metformin users. This observation indicates that whole-metagenome will contain a higher proportion of genes that are involved in LPS synthesis, sulfate metabolism, and hydrogen production metabolic pathways. The growth of

proportion of these bacterial pathways could be a cause of gastrointestinal side effects that are associated with metformin therapy [Liang et al., 2005; Carbonero et al., 2012; Forslund et al., 2015].

To efficiently reach the aims of the therapy and reduce possible side effects, detailed data of metformin-induced alteration in gut microbiome is necessary to develop personalized algorithms, that also include gut microbiome composition modifications by prebiotics and probiotics. The effect of probiotics and prebiotics in combination with metformin therapy is proved to be positive in first experiments with animals [Stenman et al., 2015; Zhang et al., 2015].

STUDY GOALS AND OBJECTIVES

Study goal: to perform a wide-spectrum study on action mechanisms of antidiabetic agent metformin by analyzing blood cell derived genetic material and gut microbiome samples.

Primary objective: To evaluate the pharmacodynamic effects in healthy individuals induced by seven-day long metformin therapy.

Secondary objectives:
1) Evaluate gut microbiome taxonomic profile in association of metformin tolerance;
2) Distinguish the changes in gut microbiome induced by metformin administration from those initiated by diet;
3) Evaluate changes in white blood cell RNA expression and DNA methylation profiles;
4) Analyze the association between metabolite profile changes in blood/faeces and the gut microbiome composition;
5) Determine the effect of metformin pharmacokinetics on gut microbiome composition and DNA methylation profiles.

STUDY DESIGN

The study is interventional and longitudinal; each participant serves as a control for itself. Advantages of longitudinal study: automatic control of constant influencing factors (participant's genotype, gender) and control of constant factors arose during the study period (participant's age, socioeconomic status, environment, etc.). The individual predisposition to a specific drug-induced response of the body, is taken into account [Gilbert et al., 2016; Petersen et al., 2016].

One of the main drawbacks for cross-sectional studies based on classical case / control design is the lower ability to distinguish between the causal relationship between associated factors [Gilbert et al., 2016]. In addition, the statistical power of cross-sectional studies is negatively

influenced by high variability of microbiome composition and epigenetic patterns among individuals [The Human Microbiome Project Consortium, 2012]. Prospective studies of interactions between metformin and gut microbiome taking into account the gut microbiome profile before the use of the drug, allowing more accurate judgment of its effects, have not been performed so far.

Study group

The study will enroll 50 healthy volunteers of European descent (both sexes) at 18-64 years of age, meeting predefined inclusion/exclusion criteria. Recruitment of the healthy volunteers and further collection of biological samples will be carried out in cooperation with the Genome Database of the Latvian Population (LGDB). Only healthy participants will be involved in the study to avoid potential effect of contributing factors such as the use of other antidiabetic drugs or antibiotics, which may have a systemic effect on microbial composition and epigenetic gene regulation causing biased results. The pre-selected age range of the participants in the study (18-64 years) is defined based on the need for homogeneous microbial compositions among individuals [Claesson et al. 2011; Zapata and Quagliarello 2015]. The number of subjects in the study group is defined on the basis of the calculations of the statistical power of the study (at least 80%), taking into account the potential fluctuations within the size of the study group (≈2%), when subjects discontinue the participation in the study for non-research reasons. The confidence level used is $\alpha = 0.05$.

Involvement of participants, motivation

Information about the opportunity to voluntarily participate in the study will be published on the website of the Latvian Biomedical Research and Study Center (LBMC) (http://www.biomed.lu.lv/). Interested subjects will be invited to contact the investigator electronically or by phone to agree on their first visit, followed by procedures conducted in accordance with the study protocol.

The recruitment will not address specific groups of people, although the possibility that unprotected subjects will apply for participation in the study is not excluded, such as members of the hierarchical structures related to the industry, students and employees of the institutions subordinated to it. In order to assess the objectivity of the motivation of volunteers, the investigator or his representative will verify the free will of the interested person to participate in the study, assess the potential conflict of interests, and will inform the subjects that refusal to attend or withdrawal from the study will not lead to negative sanctions against them.

For participation in the study, volunteers are not expected to pay and will not be compensated for the expenses related to the study process (e.g., transport). Participation in the study is not directly related to personal benefit. Participation in the project is free of charge, and participants will have the opportunity to receive the results of blood tests, serving as the main motivation to fulfill the research requirements.

Inclusion and exclusion criteria

PRINCIPAL INCLUSION CRITERIA

1. Healthy person with no known illnesses at the time of application that could possibly alter the results of the study;
2. Body characteristic parameters (eg. weight) are within the conventional range;
3. Mental condition allows a person to understand the research process and give a legal consent for the participation in it;
4. Age: 18 – 64 years;
5. European descent;
6. Both women and men with reproductive potential correspond to the contraceptive requirements stated in the protocol;
7. Prior to the study-related procedures, the consent of a person's participation in the clinical study is received by submitting a signed and dated informed consent document.

PRINCIPAL EXCLUSION CRITERIA

1. Allergies to any of *Metforal* 850mg components;
2. Usage of any other medicament which is not compatible with metformin;
3. Pregnancy or lactation;
4. Type 1 or type 2 diabetes, pancreatogenic diabetes, impaired glucose tolerance (assessed by biochemical indicators in blood samples: HbA1c)
5. Polycystic ovarian syndrome;
6. Chronic intestinal tract, oncological or autoimmune diseases;
7. Renal failure or renal impairment (glomerular filtration rate anomalous by Cockcroft-Gault method);
8. Hepatic impairment (ALT measurements anomalous) or alcoholism;
9. Acute conditions with the potential effects on kidney:

a. dehydration,
b. severe infection,
c. shock;

10. Acute or chronic disease which may cause tissue hypoxia:

a. cardiac or respiratory failure,
b. recent myocardial infarction,
c. shock;

11. Diarrhea during the past week;
12. Long term previous administration of metformin;
13. Use of the following products:

a. antibiotics,
b. probiotics tablets / capsules,
c. proton pump inhibitors (eg, omeprazole (Gasec Gastrocaps, LOMAC), lansoprazole, pantoprazole, etc.),

d. Immunosuppressive agents (e.g., methotrexate, etc.),
e. Corticosteroids (e.g., cortisone, hydrocortisone, prednisolone, etc) during the previous two months;
14. Intravascular administration of iodinated contrast agents intended during the active period of clinical trial.

Figure 1. The plan of the study

<table>
<tr><td rowspan="8">Introduction to the study; Signing an informed consent form</td><td colspan="13">Period (days)</td></tr>
<tr><td>1</td><td>2</td><td>3</td><td>4</td><td>5</td><td>6</td><td>7</td><td>8</td><td>9</td><td>10</td><td>11</td><td>12</td><td>13</td></tr>
<tr><td colspan="3">Verification period</td><td colspan="7">Use of investigational drug</td><td colspan="3">Observational period</td></tr>
<tr><td colspan="12">Filling of diet diary</td><td></td></tr>
<tr><td></td><td></td><td>Blood samples collected (fasting state)</td><td>Blood samples collected (fasting state)</td><td rowspan="2">Stool samples collected</td><td></td><td></td><td></td><td></td><td></td><td>Blood samples collected (fasting state)</td><td></td><td rowspan="2">Phone call</td></tr>
<tr><td></td><td></td><td>Stool samples collected</td><td>Blood samples collected (after 10h)</td><td></td><td></td><td></td><td></td><td></td><td>Stool samples collected</td><td></td></tr>
<tr><td></td><td></td><td></td><td colspan="7">Metformin twice a day/2x850mg</td><td></td><td></td><td></td></tr>
<tr><td colspan="3">General questionnaires filled</td><td colspan="7"></td><td colspan="2">Questionnaires of side effects filled</td><td></td></tr>
</table>

Figure 2. Flow diagram of the study design

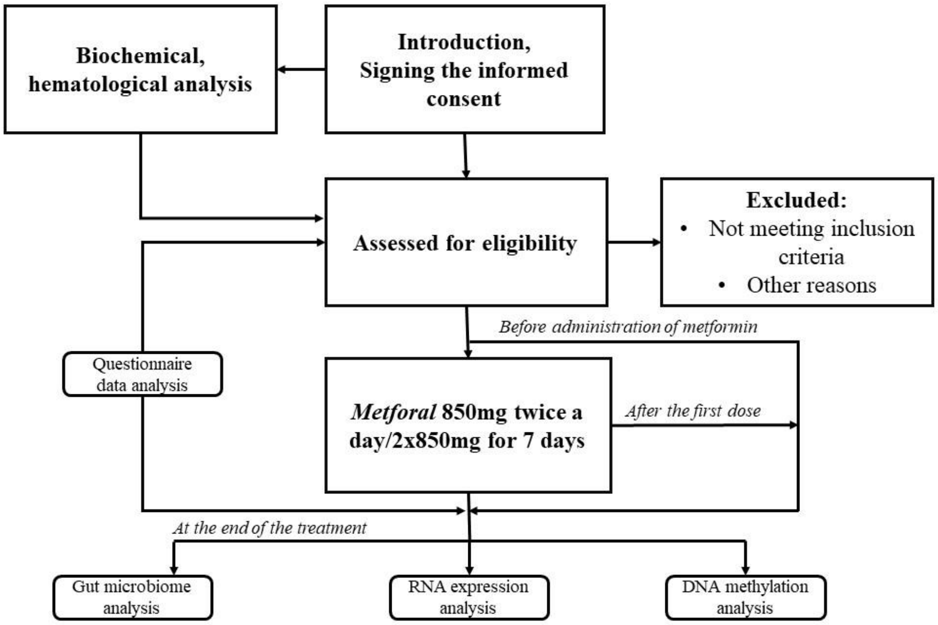

Study periods, duration of participation

Recruitment of volunteers will be organized in small groups to ensure (1) a higher adaptation of the study period to the participants' daily schedule, (2) more effective and easier sampling and immediate processing of the obtained biological samples. The study will consist of 3 periods (Figure 1, Figure 2):

1. Verification period

Subjects who will give their consent to participate in the trial by signing the informed consent form and meet the inclusion/exclusion criteria of the study, will further receive a participant's set. The participant's set includes a general questionnaire, questionnaire on health and heredity developed by the LGDB, a copy of an informed consent document, a questionnaire on observed side effects, diet diary, tubes for microbiological sampling and microbial sampling protocol (translated questionnaires are available upon request). The participant's set includes all the necessary instructions, materials and documentation forms to independently conduct the activities intended during the clinical trial. During the verification period hematological and biochemical analysis will be conducted in order to evaluate the health status of the volunteer.

2. Use of investigational drug

Only the volunteers whose biochemical analysis and the information filled in the general questionnaire still indicate on a compliance with the inclusion / exclusion criteria will continue to participate in the study. The study participants will use film-coated tablets of *Metforal* 850 mg twice daily (within the interval of 10 to 12 hours) for 7 days. In parallel, participants will be asked: (1) to document their nutrition in a previously issued diet diary on a regular basis, (2) to collect stool samples independently at home and (3) to donate blood samples on their scheduled visits.

During the study, participants will be asked to refrain from drastic lifestyle alterations and dietary changes, except for the activities required for the study.

3. Observational period

After a seven-day use of *Metforal* 850 mg, the participants will fill out the questionnaire on the observed side effects assessing the feeling of well-being during the study. The day after the last dose of *Metforal* 850 mg, the last blood and fecal sample will be collected.

Participants will be involved in the study for a duration of 13 days. Within the framework of the study seven visits to the investigator and one phone visit will be organized:

1st visit. During the first visit the investigator will introduce the potential study participant with the course of the study, inform about possible risks and benefits and provide the informed consent form. After this visit, each potential participant will get acquainted with the informed consent form and make a decision on participation in the study.

2nd visit - Study Day 1. Volunteers agreed on their participation in the study will submit a signed and dated informed consent form to the investigator. The investigator will evaluate the participants' compliance with the inclusion / exclusion criteria. In case of non-compliance, the reasons for the exclusion of the participant will be documented. During the visit, participants will receive a copy of the informed consent form signed by the participant and the investigator, instructions for stool sample collection, tubes for stool sample collection, diet diary form, general questionnaire, questionnaire on side effects, LGDB health and heredity questionnaire. During the visit, the investigator will carry out the biometric measurements necessary for the completion of LGDB questionnaire. Starting from this day, the participant will be asked to fill out the diet diary in paper or electronic format following the instructions given therein. During the verification period (see Figure 1), the participant will be asked to collect their first stool sample for the gut microbiome analysis, before starting the administration of metformin. The collection and storage of stool samples will be carried out in accordance with the procedure described in the stool sampling protocol. The participant will be asked to find the nearest E. Gulbis laboratory where venous blood samples (11 ml) for hematological and biochemical analysis will be collected. After receiving the results of hematological and biochemical analysis the investigator will evaluate the compliance of the study participant again.

3rd visit - Study Day 4. Blood samples (7 - 13 ml) will be collected from the participant's at fasting-state for further genotyping, DNA methylation, RNA expression and metabolite analysis. Participants will deliver a filled general questionnaire previously collected stool samples. The investigator will provide each participant with 14 film-coated *Metforal* 850 mg tablets and re-instruct on the mode of administration, duration, dose, storage and return of the drug. The administration of *Metforal* 850 mg will begin.

4th visit - Study Day 4. 10 hours after the first dose of the *Metforal* 850 mg (before the second dose), blood samples (7 - 13 ml) from the study participant will be repeatedly collected for DNA methylation, RNA expression and metabolite analysis. Participants will be instructed to collect the second stool sample on day 5 of the study.

5th visit - Study Day 6. Participants will be asked to attend a control visit in order to obtain the information about the participant's well-being and evaluate their compliance with the study protocol. Participants will bring the stool samples collected on Day 5.

6th visit - Study Day 11 (the day after the last intake of *Metforal* 850mg). Blood samples at fasting-state will be obtained for DNA methylation, RNA expression and metabolite analysis. If necessary, the participant will return the unused *Metforal* 850 mg tablets. Participants will be asked to collect a stool sample within this day.

7th visit - Study Day 12, The final inspection visit. Participants will bring the stool samples collected on Day 11, filled diet diaries and questionnaire on side effects. The participant of the study will be informed about the upcoming telephone call on the 13th day of the study.

Phone call visit - Study Day 13. The investigator will contact each participant by phone, making sure about the participant's well-being and any side effects observed. If necessary consultation of physician or other certified specialist will be recommended. The investigator will answer any question regarding the progress of the study and inform the participants about the end of the active participation in the study.

Premature withdrawal of subjects

During any study period, the participant has the rights to terminate the participation in the study and to prohibit the analysis of any collected data or samples. It is considered as a withdrawal of the study, therefore the investigator must be informed.

In case of premature withdrawal, the participant will return the unused *Metforal* 850 mg tablets, provide filled questionnaires and collected stool samples to the investigator. In a case-specific manner the investigator will agree to participant on further scientific application of the results obtained from the participant during the study. Based on the observations made about the participant's health, the need for a visit to a physician or certified specialist will be considered. On the 13th day of the study the investigator will re-examine all the study subjects during in the form of phone call visit. Withdrawn subjects will not be replaced with other volunteers.

Reasons for withdrawal:

1. Moderate or severe adverse drug reactions observed.
2. Use of other medications that are incompatible with *Metforal* 850 mg or may affect the results of the study.
3. Other conditions essential for the subject.

Cases where a participant of a study is excluded from participation in a study following a sponsor or researcher's decision:

1. Incompliance with the activities specified in the protocol: lack of one or more blood or stool samples.
2. Use of undesirable medications (antibiotics, probiotics) during the trial, which may affect the study results.

METHODOLOGY

Investigational drug

Metformin (*Metforal* 850 mg, Registration No. 00-1081, Anatomical Therapeutic Chemical (ATC) code A10BA02) is an oral antidiabetic drug with antihyperglycemic activity. *Metforal* 850 mg contains the active substance metformin. It belongs to a group of medicines called biguanides for the treatment of type 2 diabetes mellitus (non-insulin-dependent diabetes) in adults and in children of 10 years of age or in children and adolescents above 10 years. Metformin is a dimethylbiguanide that lowers both basal and postprandial plasma glucose levels. It is used when diet therapy and exercise do not provide adequate glycemic control (summary of drug characteristics, harmonized SAM of Latvia 30.06.2011). Metformin is recommended as a first-choice medicine to prevent hyperglycemia in patients with type 2 diabetes (T2D) [International Diabetes Federation Guideline Development Group, 2014; Inzucchi et al., 2015]. In addition, the drug may be used as a prophylactic agent in patients with impaired glucose tolerance in order to reduce their health status deterioration and the risk of developing diabetes mellitus [Canadian Diabetes Association Clinical Practice Guidelines Expert Committee, 2013; Hostalek et al., 2015; Inzucchi et al., 2015]. The benefits of metformin therapy are: high safety profile (low risk of hypoglycemia (0-21%) compared to other oral antidiabetic medication products), high efficacy, neutrality or lowering effect on body weight and cardioprotective effects [Bolen et al., 2007; Fung et al., 2015; Inzucchi et al., 2015].

Three antihyperglycemic mechanisms of action have been observed for metformin: suppression of gluconeogenesis and glycogenolysis, improvement of peripheral glucose uptake and use, inhibition of absorption of glucose in the intestine (summary of drug characteristics, harmonized SAM of Latvia 30.06.2011). Adenosine monophosphate activated protein kinase (AMPK) is considered to be the dominant mechanism underlying metformin action at the molecular level [Zhou et al., 2001; Musi et al., 2002; Diamanti- Kandarakis et al., 2010]. AMPK, the cell's energy sensor, is involved in many metabolic processes by regulating enzymes that provide gluconeogenesis, lipid synthesis and oxidation [Lan et al., 2008; Canto et al., 2009; Cao et al., 2014]. AMPK stimulates translocation of the glucose transporter 4 (GLUT4) to cell membranes, partly explaining higher uptake of glucose in tissues in the presence of metformin (Lee et al., 2012). Functional mechanisms of the drug also cover the activation of classic insulin signaling pathway (IRS/PI3K/Akt) [Xu et al., 2016]. Inhibition of mitochondrial complex I changes the ATP/AMP ratio and secondary activates AMPK, reduces the activity of mitochondrial glycerophosphate dehydrogenase (mGPD), which blocks glucose synthesis from glycerol [Andrzejewski et al., 2014; Madiraju et al., 2014; Hur and Lee, 2015].

The bioavailability of orally taken metformin ranges from 50 to 60%. The medication is absorbed in the small intestine and further directed through circulation to target organs, with the highest concentration reached in the liver (3-5 times higher than in the gate vein). Metformin is not metabolized in the body and is extracted unchanged through the kidneys [Graham et al., 2011; McCreight et al., 2016]. After an oral dose intake, 20-30% is not absorbed and is excreted in the feces. The liver is considered to be the primary target organ of metformin and its glucose lowering

effect is mainly due to the reduction of gluconeogenesis in hepatocytes [Hundal et al., 2000]. Other effects include the promotion of glucose uptake, inhibition of lipogenesis and activation of fatty acid oxidation in the liver, muscle and adipose tissues, and inhibition of lipolysis in adipose tissues [Diamanti-Kandarakis et al., 2010].

Dosage, packaging, storage of the investigational drug

Each study participant will take 850 mg of metformin twice a day (10 to 12-hour interval) for a week. During the study, the therapeutic daily dose of the drug (1700 - 2250 mg) will not be exceeded. Participants will use the medication during or after eating with a glass of water to reduce the risk of side effects. Each study participant will receive a package of 14 *Metforal* 850 mg tablets during their 3rd visit. Participants will be instructed on the dosage, regimen, duration and possible side effects of the medication. According to the manufacturer's instructions, the product does not require any special storage conditions. The drug will be delivered to the research participant in its original package - PVC / Aluminum plate.

Administration of investigational drug

Participants of the study will orally use *Metforal* 850 mg coated tablets twice daily for 7 days. The use of the drug will take place without a direct supervision of a doctor or investigator, although the compliance with the protocol will be controlled within regular visits. 14 *Metforal* 850 mg tablets will be delivered to the participants in its original package by the investigator or his representative during the 3rd visit, the participants will receive instructions for the appropriate use of the investigational drug, the number of tablets delivered to participants as well as the date of delivery will be strictly documented.

Blood sample collection

Within the framework of the study, venous blood samples (7 - 13ml) will be collected from participants (1) during the verification period, (2) immediately before the use of metformin, (3) 10 hours after the first dose of metformin, (4) 7 days since the beginning of metformin administration. During the verification period, the participant will donate a blood sample in any E. Gulbis laboratory (11 ml) for hematological and biochemical analysis. Blood samples for the analysis of DNA, RNA and metabolites will be collected on day 4 of the study (before and after the first dose of metformin) and on day 11 at LBMC. Venipuncture will be performed by a certified medical practitioner using appropriate equipment.

Hematological and biochemical analysis

The hematological and biochemical analysis will be performed in a specialized laboratory in accordance with generally accepted standard procedures. The analysis will include the following measurements: total blood count, vitamins (vitamin B12, etc.), creatinine, HbA1c, plasma glucose, bile acid, total bilirubin, ALT, GGT, alkaline phosphatase, CRO, total cholesterol, non-ABL cholesterol, high / low density cholesterol and triglycerides, etc. The list of analysis to be

performed can be changed based on the results obtained and according to the direction of the research progress.

DNA and RNA analysis

Genomic DNA from venous blood samples will be extracted according to a standard phenol-chloroform extraction technique in a LGDB laboratory. RNA isolation will be done with commercially available reagent kits such as the 5Prime PerfectPure RNA Blood Kit, following the manufacturer's instructions. DNA and RNA samples will be stored at -70 ° C in the LGDB premises.

The DNA extracted from blood samples (obtained in the 3rd visit) will be further used for genotyping in order to identify the polymorphisms so far associated with metformin tolerance, which will facilitate the selection of participants who are genetically predisposed to experience adverse reactions during metformin use. The genetic analysis will provide more information about the genetic factors of the host potentially affecting the gene expression profiles, composition of the gut microorganisms and their functions.

In addition, metformin-induced changes in the epigenetic gene regulation, DNA methylation and gene expression profiles, will be determined by comparing reference samples (collected before taking the first metformin dose on visit 3) with samples obtained after the first dose of *Metforal* 850 mg (10 h after the first metformin dose, visit 4) and samples collected at the end of *Metforal* 850 mg treatment for 7 days (visit 7). DNA methylation analysis will be adjusted by neutrophil/leukocyte ratio, which will be calculated using hematological parameters. Measurements of metformin concentration in plasma will be used as an additional factor in DNA methylation analysis. The functionality of the alterations identified in the DNA methylation analysis will be confirmed based on the correlation between the DNA methylation profiles and RNA expression level at specific loci. An association between DNA methylated profiles, transcriptome profiles and metformin tolerance indicators will be analyzed. These findings will provide information on the molecular mechanisms underlying the multiple therapeutic directions of metformin.

Methods: For global DNA methylation analysis bisulfite conversion will be performed (EZ DNA Methylation-Gold kit, Zymo Research). Next generation sequencing of bisulfite converted DNA (Illumina 450K Methylation Microchip, Illumina iScan system) is planned as an outsourcing at Uppsala University, Sweden. RNA-Seq will be performed using Low Input RiboMinus Eukaryote System v2, Ion Total RNA-Seq Kit v2 (Thermo Fisher Scientific) on Ion Proton sequencer, Ion PI Chip in LBMC. Genotyping as outsourcing will be done with Infinium Core-24 Kit, Illumina and Illumina iScan system.

Stool sample analysis

The study participants will collect the stool samples by using specific collection tubes at three consecutive time points: before administration of metformin, on the day when the treatment is started (after the first dose) and after a week-long metformin treatment. During the

regular visits, the investigator or his representative will advise the participants about an optimal time for collecting stool samples. Participants will deliver the samples to LBMC during their regular visits. Samples will be collected in two aliquots, one of them will be used for the isolation of microbial DNA and subsequent metagenome and metabolite analysis, the second aliquot will be stored as a backup sample at -70 ° C in the BMC premises.

The microbial DNA will be isolated from the stool samples and further used to determine the taxonomic alterations in the composition of gut microbiome according to the 16S rRNA gene fragment sequences obtained. In addition, shotgun metagenome sequencing will be performed, providing the information about the metabolic processes and function of the gut microbiome. The alterations in the composition of gut microbiome induced by metformin will be evaluated by comparing the microbial composition of stool samples collected before the first intake of metformin and during or after administration of metformin.

Methods: Microbial DNA from fecal samples will be isolated using FastDNA kit for Soil (MP Biomedicals), following the manufacturer's instructions. The 16S rRNA gene fragment analysis and shotgun metagenomic sequencing (Ion Plus Fragment Library Kit, Thermo Fisher Scientific) will be performed on a large-scale parallel sequencing platform (Ion Torrent). A detailed analysis of the individual taxonomic groups of bacteria will be carried out using the Sanger sequencing method and real-time PCR. Determination of metformin and various metabolites in samples (blood and stool) will be carried out with MALDI-TOF mass spectrometers and equipment available in the Latvian Institute of Organic Synthesis (OSI): gas chromatography (FID, ECD, TCD, MSD) and liquid chromatography - tandem mass spectrometry (LC-MS / MS).

The technical equipment needed to conduct the sample analysis in LBMC: automatic pipetting, PCR devices, real-time PCR devices (7500 and ViiTM7), sequencing analysis system 3130 XL, IonTorrent PGM and IonProton sequencer, MP FastPrep based on large-scale parallel sequencing technology 24 disintegrators, ultrasonic disintegration system "Covaris S220 AFA", Qubit® 2.0 fluorometer and Agilent 2100 bioanalyzer for measuring DNA quality.

Questionnaires

During the second visit the study participants will receive a diet diary and a general questionnaire. The general questionnaire includes questions related to the participant's health status and nutrition. The information gathered will be further used to identify additional factors influencing the composition of gut microbiome and epigenetic profiles. General information on health and heredity will be collected as a routine procedure in LGDB. The diet diary contains clear instructions for accurate description of the daily diet and assessment of the level of physical activity during the study. The diet diary will be provided in both printed and electronic forms. At the end of the study participants will be asked to fill the questionnaire on side effects, assessing the feeling of well-being during the study period and documenting the side effects observed. Participants will be requested to immediately inform the organizers in case of using

other medications during the trial period, in order to identify the health conditions unrelated to metformin.

Evaluation of efficiency of investigational product

The metformin-induced effect will be evaluated based on the results of microbial, metabolite, pharmacokinetic, DNA methylation and RNA expression analysis.

Primary parameters for efficacy measurements:
1. Alterations in the composition of the gut microbiome of healthy individuals (number of taxonomic units, proportion);
2. Alterations in the functions of gut microbiome in healthy individuals (number of gene families represented, signaling pathways, metabolites and their concentrations);
3. Alterations in DNA methylation and transcriptome profiles (Beta value, fold-change, Spearman correlation coefficient, p-value).

Secondary parameters related to efficacy:
1. Association between specific composition of gut microbiome and the severity of the side effects (hi squared value, p-value);
2. The association between DNA methylation at specific loci, gene expression profile with severity of side effects (hi squared value, p-value);
3. The association of the composition of gut microbiome with altered DNA methylation or transcriptome profiles (hi squared value, p-value).
4. Association between pharmacokinetic parameters and specific taxonomic units, changes in DNA methylation profiles and transcriptome profiles (p-value).

The metagenome analysis will provide information on the effect of metformin on the microbial gut, its functions, and the role of microbial in metformin tolerance. The microbiological analysis will be carried out in the specimens of the feces using the methods described above. The effect of metformin on the intestinal microbial will be evidenced by the altered microbial composition and function - a statistically significant change in the number of taxonomic units, genes, proportions, identified metabolites and their concentrations.

Metagenomic analysis will provide information about the metformin-induced alterations in gut microbiome and its role in the tolerance of metformin. The metagenomic analysis will be performed by using stool samples and the effect of metformin will be evaluated by altered composition and function of gut microbiome - significantly changed number of taxonomic units, genes, proportions, metabolites and their concentrations.

Venous blood samples will be further used for DNA methylation and RNA expression analysis. The effect of metformin on the epigenetic regulation will be confirmed by statistically significant alterations in the DNA methylation and transcriptome profiles after the administration of metformin, and the functionality of these alterations will be confirmed based

on the correlation between the DNA methylation profiles and the RNA expression intensity at specific loci.

The ability of metformin to induce alterations in the composition of gut microbiome and epigenetic gene regulation will be analyzed using biological materials (venous blood and feces) derived from three treatment-related time points: before metformin use, 10 hours after the first dose of metformin and 7 days after metformin use. For each participant of the study, alterations in the diversity and proportion of microbial taxonomic units of the gut microbiome and metabolite composition and concentrations in stool samples will determined. In addition, metformin-induced alterations in DNA methylation and transcriptome profiles will be analyzed. Data obtained from biological samples collected before the use of metformin will be considered as a reference. By combining the results of all participants, conclusions will be drawn on the effect of metformin on the composition of gut microbiome and epigenetic regulation. Statistical data analysis will allow to determine an association between microbial composition and severity of adverse reactions, microbial composition and changes in DNA methylation or RNA expression profiles, DNA methylation profiles and drug tolerance.

Endpoints of the study

<u>The primary endpoint</u> of the study is a significant change in the composition of the gut microbiome in healthy subjects (composition and proportion of taxonomic units) after administration of metformin.

<u>Secondary endpoints</u> of the study:

1) Significant alterations in DNA methylation profiles and RNA expression levels in healthy subjects following metformin use;

2) Association of DNA methylation profiles with severity of adverse reactions.

3) Association of specific gut microbial taxonomic units with severity of adverse reactions;

4) The association of specific taxonomic units with changes in the metabolite profile in the blood / faeces.

5) Association of pharmacokinetic parameters with specific taxonomic units of gut microbiome and changes in DNA methylation and gene expression profiles.

SAFETY CONSIDERATIONS

The research safety assessment will be carried out during each visit based on the information provided by participant on their well-being and health status since the beginning of *Metforal* 850 mg administration or previous visits. All participants of the study will be advised to inform the research organizers by telephone on any arisen health risks also outside the time of visits, if necessary. During the study all the side effects observed will be documented in detail in a specific questionnaire, indicating the type of side effects observed, time, and frequency of recurrence. Approximately 72 hours after the last dose of *Metforal* 850 mg the investigator will survey on phone all participants involved in the study in order to obtain more

information and evaluate their state of health and, if necessary, recommend visiting physician or certified specialist.

If any of the study participants is harmed as a result of the clinical trial, the compensation will be paid in accordance with the sponsor's concluded insurance contract. Visits to the physician, in case of side effects or adverse events will be compensated after the submission of payment document and a physician’s conclusion that the cause of observed side effects is metformin therapy.

Definitions

Adverse Event (AE) or Adverse Experience - any unfavourable and unintended sign (including an abnormal laboratory finding), symptom, or disease temporally associated with the use of a medicinal product, whether or not considered related to the medicinal product.

Serious Adverse Event (SAE) or Adverse Drug Reaction - any adverse drug event (experience) occurring at any dose that in the opinion of either the investigator or sponsor results in any of the following outcomes: 1) Death 2) Life-threatening adverse drug experience 3) Inpatient hospitalization or prolongation of existing hospitalization (for > 24 hours) 4) Persistent or significant incapacity or substantial disruption of the ability to conduct normal life functions 5) Congenital anomaly/birth defect 6) Important Medical Event (IME) that may not result in death, be life threatening, or require hospitalization may be considered a serious adverse drug experience when, based upon medical judgment, it may jeopardize the patient or subject and may require medical or surgical intervention to prevent one of the outcomes listed in this definition.

AE classification by severity:

Mild - Awareness of signs or symptoms, but easily tolerated and are of minor irritant type causing no loss of time from normal activities. Symptoms do not require therapy or a medical evaluation; signs and symptoms are transient.
Moderate - Events that introduce a low level of inconvenience or concern to the participant and may interfere with daily activities, but are usually improved by simple therapeutic measures; moderate experiences may cause some interference with functioning
Severe - Events that interrupt the participant’s normal daily activities and generally require systemic drug therapy or other treatment; they are usually incapacitating

Assessment and reporting of adverse reactions

The adverse reactions observed will be documented by the participant at the end of the active period of the study by using a specific questionnaire on side effects. The investigator will immediately inform the sponsor of any serious adverse event related to the subjects involved in the clinical trial. The sponsor will ensure that all undesirable events reported by the investigator or his research team are documented. At the end of the active period of the study the investigator does not apply monitoring of undesirable events to subjects involved in the clinical trial. If the

investigator receives information about serious adverse events after the trial is completed, the investigator will report it to the sponsor.

Potential benefits and risk factors for the study participants

Participation in the study is not directly related to personal benefits of participants. Participation in the study, blood tests, genomic and microbial analysis conducted within the framework of the study are free of charge, and participants will receive the results of hematological and biochemical blood tests.

The risk factors for the participation in the study are related to the potential side effects of *Metforal* 850 mg. Based on the Summary of Product Characteristics gastrointestinal side effects are the most common (more than 1 in 10 people) in adults: indigestion, nausea, vomiting and diarrhea, abdominal pain, loss of appetite. Common (affects less than 1 in 10 people) alterations in taste, very rare (affects less than 1 in 10,000 people) skin reactions (erythema, itching and urticaria), abnormal liver function tests, hepatitis, decreased vitamin B12 blood. Very rare but serious side effect is metformin-induced lactic acidosis. Venipuncture will be performed by certified medical staff. In rare cases, a small amount of blood flushing may occur, or, less rarely, a slight localized inflammation of the skin. Collection of the stool samples does not endanger the health of the participant if general hygiene requirements are met.

In order to minimize the risk of undesirable effects, inclusion / exclusion criteria were thoroughly developed taking into account the contraindications for metformin listed in the Summary of Product Characteristics. Study participants who have moderate or severe side effects will immediately stop the use of the medication and, if necessary, will be advised to consult a physician or certified specialist. Participants will be asked to inform the investigator about the side effects observed, by documenting their observations in questionnaire on side effects at the end of the study. Participants will be informed about the risks of the study prior to the study, emphasizing their rights to discontinue the participation at any time and to inform that refusal to attend or withdrawal from the study will not result in any sanctions against them. The participant will confirm his awareness of the potential risks of the study by signing the informed consent form.

Interaction with other medications and other forms of interaction

Based on the information provided in the Summary of Product Characteristics for *Metforal* 850 mg, simultaneous use is not recommended with:

- Alcohol. Acute alcohol intoxication is associated with an increased risk of lactic acidosis, in particular: at fasting state or inadequate dietary intake; liver failure. Alcohol or ethyl alcohol containing medications should be avoided.
- Contrast medicines containing iodine. Administration of intravascular iodine contrast agents may lead to renal failure, which results in accumulation of metformin and increased risk of lactic acidosis. Metformin should be discontinued prior to the intended examination and should

be restarted at least 48 hours after the examination and re-evaluation of renal function.

Use of other medications/substances during the trial

For safety reasons participants during the study will be asked:

1. To do not use alcohol and alcohol containing medicines that increase the risk of lactic acidosis;
2. To do not use glucocorticoids (systemic and local), sympathomimetics and diuretics with internal hyperglycaemic activity;
3. To do not use angiotensin-converting enzyme (ACE) inhibitors that may lower blood glucose levels;
4. To do not use medicines transported by organic cationic transport molecules-2 (OCT2);
5. Avoid intravascular administration of iodine-containing contrast agents that may cause renal insufficiency and accumulation of metformin hydrochloride and increase the risk of lactic acidosis. If necessary, metformin will be discontinued 48 hours before the intended examination;
6. Inform the investigator about the use of any additional medicines, in order to objectively evaluate adverse reactions and study results.

In cases where the administration of metformin will harm the health status of the participant or confound the effectivity of other indispensable medications, the use of metformin and the participation of the person in the study will be discontinued. Participation in the study will be discontinued even if the participant used undesirable medications (antibiotics, probiotics) during the study, which may affect the study results. Participants will be asked to inform the investigator about the use of other drugs during regular visits and record them in the questionnaire on side effects.

Contraindications

1. Hypersensitivity to the active substance or to any of the excipients in medication (Hypromellose, Povidone K 25, Magnesium Stearate (Ph. Eur.), Hypromellose, Macrogol 6000, Titanium Dioxide (E 171)).
2. Diabetic ketoacidosis, diabetic trauma.
3. Renal impairment (creatinine clearance <60 ml / min).
4. Acute conditions with potentially impaired kidney function, such as
    a. Dehydration
    b. severe infection
    c. shock.
5. Administration of intravascular iodine contrast agents.
6. Acute or chronic disease that can cause tissue hypoxia, such as

a. heart or respiratory failure,

b. recent myocardial infarction

c. shock.

7. Hepatic insufficiency, acute alcohol poisoning, alcoholism.

Contraception requirements

Since metformin is characterized as an antidiabetic agent with unlikely teratogenicity / fetotoxicity in early pregnancy there are no contraceptive requirements for men who have partners with reproductive potential in the study.

Women of childbearing potential must initiate or continue to use the appropriate contraceptive method:

1. Combined (progestogen and estrogen-containing) hormonal contraception;
2. Progestogen hormonal contraception;
3. Intrauterine contraceptive device;
4. Hormone releasing intrauterine contraceptive device;
5. Partner with vasectomy;
6. Complete abstinence;
7. Barrier methods (male or female condom with or without spermicide).

There is no evidence of reduced contraceptive efficacy in metformin users in case of hormonal contraception. The appropriate contraceptive method should be followed throughout the study period and continued for at least 3 days after the last dose of metformin (until the product is released from the body). During the verification period of the study a pregnancy test (beta HCG) will be performed for women with reproductive potential. Additional pregnancy tests are not planned during the study.

FOLLOW-UP

The follow-up period will be 13 days in total, including three days after the last intake of investigational drug in order to record any possible adverse events arose after the active period of the trial and data collection.

DATA MANAGEMENT AND STATISTICAL ANALYSIS

Data storage and data protection

The information provided by the participant remains confidential and will not be disclosed without the consent of the participant. The sponsor in cooperation with the LGDB ensures the storage and protection of data in accordance with Latvian legislation (Personal Data Processing Law, into force from 05.07.2018.) Data acquisition, processing, verification and reporting will be carried out by experienced scientific LBMC staff. Access to data will be provided to a limited number of individuals designated by the sponsor and responsible investigator. Data collected in a printed form (questionnaires, diet diary, informed consent form) will be copied and stored for the security reasons in the LGDB premises ensuring

restricted access. Electronic data will be assigned with an authorized approach and will be duplicated on an external data storage device.

The case report form will not be used during the study. Each participant in the study will have a personal data set that will consist of summarized primary documents (general questionnaire, questionnaires on side effects, LGDB questionnaire, diet diary, results of biochemical analysis, reasons for exclusion) and will be stored in printed form. Primary data will be analyzed with specific statistical data processing programs, according to the objectives and tasks of the study. The personal data collected during the study will be stored for 3 years after the end of the study.

The biological material and phenotypic data collected in LGDB during the study will be used only for research purpose. The use of data will be managed in accordance with the restrictions specified in the informed consent form.

For security reasons, the data will be encoded with the code approved by the State Data Inspection and assigned by LGDB. Participant's personal identification data will be stored in the LGDB without access to third parties, in accordance with the Regulations of the Cabinet of Ministers of the Republic of Latvia.

The information about the participant's genotype will be entered into the LGDB and transferred to the The State Genome Registry, as required by Latvian legislation. Compensation for moral damage will be provided if sensitive data obtained during the study will be at the disposal of a third party (in accordance with the Latvian legislation, on a case-by-case basis).

Data availability to authorized persons

The sponsor and investigator will allow any direct monitoring, audit and regulatory inspection of the clinical trial ensuring an access to the primary documentation and associated data. The procedures mentioned above will be conducted in accordance to Regulations of the Cabinet of Ministers of the Republic of Latvia preserving the confidentiality of the participants in the study. By signing the informed consent participants allow their data to be accessed by persons managing the monitoring, audit and regulatory inspections.

Methodology for data statistical analysis

Most of the data within the study will be obtained by massive parallel sequencing techniques, therefore, data pre-processing and specific preparation for statistical analysis is needed (e.g., removing low quality data or data that do not correspond to statistical standards). Data analysis will be performed using such softwares as mothur v.1.39.1, R, RStudio, Galaxy and others. The applied methodology for data statistical analysis may be changed according to offered possibilities, and within the study, the used approaches will be updated to ensure usage of the most recent, best quality, and suitable software, tools, tests, and their versions.

For the gut microbiome data, the sample inner (alpha) diversity and significance of its changes will be calculated (by Shannon index, t-test), as well as performed evaluation of inter-

sample (beta) diversity (principal coordinate analysis (PCoA, R, “vegan”). The statistically significant changes within every taxonomic level will be calculated (statistical tests: t-test, F-test, GLM, regression analysis; R „metagenomeSeq”, „edgeR”, „limma”, „lme4”, „lmerTest”, „multcomp”, „rgl”, etc.).

The data on methylation and RNA expression profiles will be used to determine genomic regions and their associated pathways with significantly altered DNA methylation or RNA expression levels (statistical tests: t-test, F-test, GLM, regression analysis; R „edgeR”, „DESeq2” „asmn”, „sva”, „limma”, „lumi”, „wateRmelon”, „TCGAMethylation450k”, etc.).

Biochemical, haematological, and anthropometric characteristics will be used as covariates in the performed statistical analyses.

After collection of all data, the following associations will be assessed:

- Between abundance of specific taxonomic groups and severity of observed side effects (chi-squared test);
- Between changes in DNA methylation profile and severity of observed side effects (ANOVA);
- Between abundance of specific taxonomic units and changes in DNA methylation profile (ANOVA);
- Between pharmacokinetic characteristics and abundance of specific taxonomic groups, changes in DNA methylation profile (ANOVA).

QUALITY ASSURANCE

The sponsor is responsible for ensuring and controlling the quality of activities carried out and the data obtained within the framework of the study. The collection, transportation, processing and storage of the samples will be performed on the basis of internal standard operation protocols developed by the Sponsor (SOP), which comply with guidelines for good clinical practice and the Regulations of the Cabinet of Ministers of the Republic of Latvia.

Venipuncture will be performed by a certified medical personnel, using single use systems, ensuring full safety for patients and medical personnel, and by excluding contamination errors in the analysis performed. For the purpose of monitoring the quality of the collection of the stool samples and transfer instructions will be issued to the research participants. Storage of stool and blood samples will take place under conditions appropriate to the type of sample (observing sterility, temperature, etc.).

Biochemical and hematological analysis will be performed by E. Gulbis Laboratory certified medical personnel, in accordance with the quality requirements of LVS EN ISO 15189:2007. The analysis of microbiome, DNA and RNA will be carried out by trained personnel of the LBMC, in accordance with the analytical methods protocols developed by

LBMC. The statistical processing of the resulting data will be carried out by qualified LBMC staff on the basis of an internationally accepted methodology, adapted to the design of the study. The sponsor, in cooperation with the LGDB (certified data keeper), provides storage and protection of data in accordance with the Personal Data Processing Law (into force from 05.07.2018.). Access to data related to the progress of the study will be provided to a limited number of persons selected by the Sponsor.

Monitoring

Monitoring will be done by a sponsor-selected person having a prior scientific experience in local and international research projects. Monitoring will be performed before the beginning of the trial, during the trial and at the end of the trial. The purpose of the monitoring is to evaluate the respect of participant rights, the reliability of the reported data and their compliance with the source data, the compliance of the study process with the National legislation of Latvia, the sponsor's internal standard protocol of operations, the study protocol, and Guideline for Good Clinical Practice. Within the framework of the monitoring, the qualification of the research team and available resource, the process of delivery, storage and release of investigational drug will be evaluated. In addition, monitor will verify the time of signing the informed consent, documentation of adverse reactions, filling and storage of all the documentation related to the participant, documentation of any withdrawal. Following the monitoring, the monitor will submit a written and dated report to the sponsor summarizing the monitoring activities and observed discrepancies. The monitor will advise the investigator or his team on further actions if necessary.

EXPECTED OUTCOMES OF THE STUDY

The results of this study will improve the knowledge on metformin’s functional mechanisms in the human body. It is expected that in long-term these results will allow to make prognosis for patients about efficiency of metformin therapy and the risk of unwanted side-effects, therefore, facilitating precision medicine based decisions for treatment strategy.

DISSEMINATION OF THE RESULTS AND PUBLICATION POLICY

The findings of the study will be presented at local and international scientific conferences, as well as published in peer reviewed scientific articles, where anonymity of the participants will be ensured. Publication or reporting of study results at conferences will be coordinated by members of the clinical trial management staff, considering the contribution of each member of the research team. The final report of the study will be prepared by the responsible investigator and research team at the LBMC.

DURATION OF THE PROJECT

Duration of the project depends on the efficiency of recruitment process. Recruitment of volunteers will be organized in small groups to ensure a higher adaptation of the study period to the participants' daily schedule, more effective and easier sampling and immediate processing of the required samples. The recruitment will be continued until the desired number of participants is reached. Sample analysis will be conducted in multiple batches according to recruitment process and the final integrative data analysis will be conducted after the desired number of participants is reached and sample analysis performed. The study might be completed in year 2020, although the duration of the project is highly depending on recruitment efficacy.

PROBLEMS ANTICIPATED

**Deviation from the protocol**	**Action of participant**	**Action of investigator**
The participant skips an intake of *Metforal* 850mg tablet.	A double dose is not used. The deviation is fixed in the diet diary.	Deviations from the protocol are taken into account during data processing.
Participant postpones the collection of stool sample.	Participant informs the investigator, marks the collection tube according to the date of sample collection, continues collection of remaining samples according to the study protocol.	The specific stool sample is excluded from the gut microbiome analysis.
Participant postpones the delivery of stool sample.	The sample is delivered to BMC as soon as possible.	Deviations from the protocol are taken into account during data processing.
Participant is not able to attend a blood donation visit in BMC.	Participant informs the investigator. If possible, the participant donates a blood samples in E. Gulbis laboratory.	Ensures sample delivery to BMC and further processing according to the protocol.
Participant is not able to attend a blood donation visit in BMC or E. Gulbis laboratory.	Participant informs the investigator, continues to donate the remaining samples according to the study protocol.	The specific blood sample is excluded from the DNA methylation and gene expression analysis.

ETHICS

The study will be conducted in accordance with the Declaration of Helsinki developed by the World Medical Association and the Oviedo Convention for the protection of human rights and dignity of the human being in biology and medicine developed by the Council of Europe. During the research the Cabinet of Ministers of the Republic of Latvia No. 289 of 23 March 2010 “Regulations provide for the procedure of clinical trials of medicines in conformance with the good clinical practice requirements, the procedure of observational studies of administration of medicines, marking of investigational medicinal products, as well as the procedure for assessment of conformity to the good clinical practice requirements” will be adopted and all activities within the framework of the study will be carried out in accordance with the principles of Good Clinical Practice. The research protocol has been approved by the Central Medical Ethics committee of Latvia (No. 1/16-05-12) and The State Agency of Medicines of Latvia (No. 17-1723).

Patient information sheet and informed consent form

PHARMACODYNAMIC STUDY OF ANTIDIABETIC DRUG METFORMIN

Sponsor protocol code No. MIKROMET16001

Dear Sir!/ Dear Madam!

You are offered the opportunity to participate in a study carried out under the project "Molecular mechanisms for diabetes and cardiovascular complications, pharmacogenetics and new medicinal products". Before signing the consent form, we ask you to get acquainted with the following information about the objectives and procedures of the project.

Aim of the study

Metformin is the most commonly used medication to treat diabetes. In addition to the glucose lowering effect, metformin has cardioprotective, anti-cancer and anti-aging effects. Despite the widespread use of metformin, its mechanisms of action in the organism are still not clear. The project aims to carry out wide-spectrum studies of metformin mechanisms by analysing blood cells' derived genetic material and gut microbiome samples[1]. Results obtained will serve as a basis for developing tests that will allow predicting the effectiveness of metformin therapy and the risk of developing side effects in patients, making it easier to choose a personalized treatment strategy.

[1] The term "gut microbiome" is used to describe microorganisms living in the intestinal tract and their genome (genetic material).

Participation criteria

a) You are a healthy person who does not have the following diseases/health problems:
   a. Type 1 or type 2 diabetes mellitus, pancreatogenic diabetes or glucose tolerance impairment;
   b. Polycystic ovary syndrome;
   c. Chronic intestinal, oncological or autoimmune diseases;
   d. Renal failure or renal impairment;
   e. Liver impairment or alcoholism;
   f. Dehydration, severe infection or shock;
   g. Heart or respiratory failure, recent myocardial infarction;
b) You are an adult and not older than 64;
c) You are not allergic to any of the substances contained in medicinal product;
d) You are not taking any other medicines that are incompatible with metformin:
   a. Alcohol and alcohol or ethyl alcohol containing medications;
   b. Glucocorticoids, sympathomimetics and diuretics with internal hyperglycemic activity;
   c. ACE inhibitors;
   d. Medicinal products transported by organic cationic transporter-2 (OCT2);
e) Women are not pregnant and are not breast feeding;
f) In the last two months you have not taken any:
   a. Antibiotics;
   b. Probiotics in the form of tablets/capsules;
   c. Immunosuppressive medications (e.g. Methotrexate, etc.);

    d. Proton pump inhibitors (e.g. Omeprazole (Gasec Gastrocaps, Lomac), lansoprazole, pantoprazole, etc.);
    e. Corticosteroids (e.g. cortisone, hydrocortisone, prednisolone, etc.);

g) You have not had diarrhea during the last week;
h) Metformin has not been used for a long time in a past;
i) During the study any intravascular administration of iodinated contrast agents are not planned.

Procedures of the project

The estimated duration of the study is 13 days (see study plan). If you agree to participate in the study, **you will need to take *Metforal* 850mg tablets for one week twice a day (at intervals from 10 to 12 hours)**. Each tablet contains 850mg of metformin hydrochloride, which does not exceed standard doses used for treatment. The medication will be given to you at the beginning of the study (14 tablets). Metformin is recommended to use with a glass of water.

As a part of the study, you will have to arrive at 7 visits to the Latvian Biomedical Research and Study Centre, Ratsupites Street 1, Riga, during which you will deliver faecal samples, blood samples, and consult with the organisers of the study. Within the framework of this study, a total number of three faecal samples (10-15 g) and 10 blood samples (7-13 ml) will be collected from you. You will receive a participant kit that includes tubes for collecting microbiome (faecal) samples, a microbiome sampling protocol, a questionnaire for the microbiome project, a side effect questionnaire, and a diet diary.

Your eligibility for inclusion/exclusion criteria will be evaluated at the beginning of the study. You will be given a general questionnaire, a health and heredity questionnaire, a diet diary and a side effect questionnaire. The general questionnaire as well as the health and heredity questionnaire cover general questions about your health, lifestyle and nutrition. In the diet diary, following the instructions, it is necessary to accurately write down the diet used every day and to evaluate the level of physical activity. It is advisable to start fill up the diet diary the day before the collection of first microbiome samples. The diet diary will be available in both paper and electronic format. At the end of the study, you will need to complete a side effect questionnaire, describing the side effects and their frequency reported during the study.

At the beginning of the study, you will have to go to the nearest E. Gulbja laboratory, where blood samples for biochemical and haematological tests will be taken in a fasting state.

You will be asked to collect the first microbiome sample (faeces) before starting the metformin usage on the first day of the study. **Microbiome samples must be collected and stored in accordance with the procedure described in the microbiome sampling protocol.**

On the fourth day of the study, during your visit, you will need to give a blood sample in a fasting state, before the first metformin tablet is taken, for genetic and metabolite (such as fatty acid) analysis. You will have to deliver the previously collected faecal sample to the researcher or his representative. Blood samples for genetic and metabolite analysis will be taken a second time in the fourth day, during a re-visit, ten hours after the first metformin tablet was taken (before the second dose).

On the fifth day of the study (the second day after starting the metformin usage), a second stool sample should be collected, which you will deliver to the researcher or his representative during the visit scheduled for day 6 of the study.

On the 11th day of the study (after seven days of metformin administration), blood samples will be taken again in a fasting state and a third sample of microbiome should be collected. The third stool sample, together with completed diet diary and side effect questionnaire, you will deliver to the researcher or his representative during the visit on the 12th study day.

On the 13th day of the study, the researcher or his representative will contact you by telephone to evaluate your wellbeing and provide appropriate recommendations, if necessary.

The plan of the study

<table>
<tr><td rowspan="8">Introduction to the study; Signing an informed consent form</td><td colspan="13">Period (days)</td></tr>
<tr><td>1</td><td>2</td><td>3</td><td>4</td><td>5</td><td>6</td><td>7</td><td>8</td><td>9</td><td>10</td><td>11</td><td>12</td><td>13</td></tr>
<tr><td colspan="3">Verification period</td><td colspan="7">Use of investigational drug</td><td colspan="3">Observational period</td></tr>
<tr><td colspan="12">Filling of diet diary</td><td></td></tr>
<tr><td></td><td></td><td>Blood samples collected (fasting state)</td><td>Blood samples collected (fasting state)</td><td rowspan="2">Stool samples collected</td><td></td><td></td><td></td><td></td><td></td><td>Blood samples collected (fasting state)</td><td></td><td rowspan="2">Phone call</td></tr>
<tr><td></td><td></td><td>Stool samples collected</td><td>Blood samples collected (after 10h)</td><td></td><td></td><td></td><td></td><td></td><td>Stool samples collected</td><td></td></tr>
<tr><td></td><td></td><td></td><td colspan="7">Metformin twice a day/2x850mg</td><td></td><td></td><td></td></tr>
<tr><td colspan="3">General questionnaires filled</td><td colspan="7"></td><td colspan="2">Questionnaires of side effects filled</td><td></td></tr>
</table>

During the course of the study, you are asked to avoid any radical change in lifestyle and diet, except for those needed in the study. If any health problems arise during the study and it is necessary to start a specific therapy, such as antibiotics, or if you want to withdraw from the study for other reasons, please inform the organisers of the study. **The participant under no circumstances should postpone his/her therapy due to the study** and, if necessary, can withdraw from the study.

Genetic testing. Genetic material (deoxyribonucleic acid - DNA and ribonucleic acid - RNA) will be isolated from your blood samples. Genetic testing will identify inherited changes

that may increase the risk of adverse reaction to metformin. Analysing samples taken at different time points before and after the use of the medication, the metformin induced changes in gene[2] regulation will be determined. Genetic testing will allow to clarify the molecular mechanisms of metformin. The results of genetic testing will be available to you on a written request addressed to the Genome Centre.

The genetic material obtained during the study and all related information will be transferred to the Genome Database of the Latvian Population, the aim of which is to accumulate information regarding heredity carriers of Latvian residents, or genes and environmental factors that affect disease development, in a single database. Your privacy will be maintained in accordance with the "Human Genome Research Law" and the "Privacy Data Protection Act".

[2]Gene – a heredity factor whose function is to determine the specific properties of the body.

Possible risks

Metformin is an oral antidiabetic medication that does not directly affect blood glucose levels, therefore, the risk of hypoglycaemia is minimal. The most common side effect of metformin (1/10) in patients are gastrointestinal (abdominal distension, diarrhea, nausea, vomiting), a feeling of taste may change. In some cases, skin reaction may occur, such as erythema, pruritus, urticaria. Very rare (1:33000 patients) is a risk of lactic acidosis, which is a health threatening condition.

Lactic acidosis is characterized by acidotic dyspnoea, abdominal pain and hypothermia, followed by coma. If metabolic acidosis is suspected, metformin administration should be discontinued and the patient should be hospitalized immediately. To reduce the risk of lactic acidosis during the study, we strongly recommend that you take metformin as prescribed in the study, avoid prolonged fasting, excessive alcohol intake, and intravascular administration of iodine containing contrast agents.

In case of metformin related side effects, the participant immediately stops taking the medication and, if necessary, contacts a family doctor. Presenting a payment-supporting document and the conclusion of the family doctor on the occurrence of a health disorder, related to the use of the investigational medication, **the costs associated with the treatment will be compensated by the means of the project.**

A blood sample from the vein will be taken by a trained medical practitioner, as in the usual blood test. The venepuncture site may rarely result in small bruised area or, more rarely, a small local inflammation of the skin. Collecting faecal samples does not endanger your health if you meet general hygiene requirements.

Voluntary participation

Your participation in this study is voluntary.

Confidentiality of data and rights of the study participant

Your participation in this study as well as the information provided by you, will remain confidential and will not be disclosed without your consent. All submitted samples and your completed questionnaires will be assigned a code to ensure the confidentiality of your data. The "Human Genome Research Law" as well as the "Privacy Data Protection Act" adopted by the Saeima of the Republic of Latvia, guarantees the confidentiality of your personal data, health and

heredity data, as well as any information derived from genetic research. Information will be stored at a place with limited availability and will not be issued to your family members, insurance companies, or employers. The data and tissue samples obtained will not be used for commercial purposes or illegally transferred to non-participating third parts. The personal data obtained during the study will be maintained for 3 years after the conclusion of the research. Genetic material and related information in the Genome Database of Latvian population will only be used for research purposes for an unlimited period of time. Study sponsor – Latvian Biomedical Research and Study Centre will do everything it can to preserve the confidentiality of personal data.

The data obtained during the study will be available for:

1. The principal investigataor and the personnel involved in the clinical trial;
2. The institutions involved in the study: APP "Latvian Biomedical Research and Study Centre", "E. Gulbja Laboratory", the Biomedical Centre of the University of Uppsala (with your consent), Genome Database of Latvian population;
3. Clinical trial related monitoring, audit and regulatory inspections.

In case you agree to submit your tissue samples and the description of your state of health for dispatch outside of Latvia for research purpose, other regulations may relate to the security of personal data.

Involved persons have the right to withdraw their participation at any time. Refusal or withdrawal from the study will not result in any penalties against you.

Potential benefits

Participation in the study is not directly related to personal benefit and does not entail any remuneration. Expenses related to participation in the study (transport, meals, and other expenses) will not be compensated.

The results of the project will promote understanding of metformin's mechanisms of action and factors that affects adverse reactions. The information obtained will help in developing personalized treatment algorithms. Participation in the project and the analysis provided in the project is free of charge. You will have a chance to get the results of your blood tests.

Research results

Data from the study may be presented at local and international scientific conferences, as well as published in scientific articles. If your data is published, your anonymity will be secured.

Source of funding for the study

The project is funded by National Research Program "Biomedicine for public health" project No.2 "Molecular mechanisms of diabetes and cardiovascular complications, pharmacogenetics and new medical products" and European Regional Development Fund project No. 1.1.1.1/16/A/091 "Investigation of interplay between multiple determinants influencing response to metformin: search for reliable predictors for efficacy of type 2 diabetes therapy".

Contacts

If you are experiencing any adverse reaction while taking this medication, please report this to the researcher:

Ineta Kalnina
E-mail: ineta@biomed.lu.lv
Tel.: +371 67808200

If you have additional questions about this study, please contact the Genome Centre at the **Genome Database of Latvian population:**

Tel.: +371 67473083
E-mail: gec@biomed.lu.lv
Address: Latvian Biomedical Research and Study Centre, Ratsupites Str. 1 k-1, Riga, Latvia, LV-1067.

Central Medical Ethics Committee:

Vents Silis
E-mail: vents_silis@yahoo.com

The State Agency of Medicines of Latvia:

Tel.: +371 67078424
E-mail: info@zva.gov.lv
Address: Jersikas Str. 15, Riga, Latvia, LV-1003

Code

Consent form

I confirm that I have read and understood the information provided by the information page about study on the mechanisms of metformin action, under the project "Molecular mechanisms of diabetes and cardiovascular complications, pharmacogenetics and new medical products ".

Researcher ____

Have explained to me the information about the goal and procedures of the human gut microbiome research project.

I was given the time and opportunity to carefully read this information and decide independently whether to participate in this study.

I agree to participate in this project voluntarily and free of charge.

I AGREE THAT MY SAMPLES OF TISSUES AND GUT MICROBIOME (FECES) WILL BE USED IN STUDIES CONDUCTED WITHIN THE FRAMEWORK OF THE PROJECT.

Sending my tissue samples and the description of my state of health for genetic research outside of Latvia (please mark):

☐ I allow;
☐ I forbid.

I declare that I agree to further communication, if such a need arises during the study.

I agree to participate in the study.

Participant's name, surname
(in block letters) ____

Contact information

Address: ____

____

Telephone number: ____

E-mail: ____

Signature of the participant ____________________date__________________________
(yy.mm.dd., dated by patient)

Researcher's name, surname (in block letters)______________________________

Signature of the researcher: ______________________date_______________________

Part 2

COLLABORATION WITH OTHER SCIENTISTS OR RESEARCH INSTITUTIONS

1) E. Gulbis laboratory - hematological and biochemical analysis;
2) Uppsala Biomedical Centre, Uppsala University – DNA methylation analysis;
3) Latvian Institute of Organic Synthesis – metabolite analysis.

FINANCING AND INSURANCE

The project is funded by National Research Program "Biomedicine for public health" project Nr.2 "Molecular mechanisms of diabetes and cardiovascular complications, pharmacogenetics and new medical products" and European Regional Development Fund project Nr. 1.1.1.1/16/A/091 " Investigation of interplay between multiple determinants influencing response to metformin: search for reliable predictors for efficacy of type 2 diabetes therapy".
The insurance contract is concluded with the local insurance company “Balta” (https://www.balta.lv/lv) as Professional Civil Liability Insurance.

REFERENCES

Andrzejewski, S., Gravel, S.P., Pollak, M. and St-Pierre, J. Metformin directly acts on mitochondria to alter cellular bioenergetics. *Cancer Metab* **2** (2014), p. 12.

Barres, R., Yan, J., Egan, B., Treebak, J.T., Rasmussen, M., Fritz, T., Caidahl, K., Krook, A., O'Gorman, D.J. and Zierath, J.R. Acute exercise remodels promoter methylation in human skeletal muscle. *Cell Metab* **15** (2012), pp. 405-11.

Barres, R., Kirchner, H., Rasmussen, M., Yan, J., Kantor, F.R., Krook, A., Naslund, E. and Zierath, J.R. Weight loss after gastric bypass surgery in human obesity remodels promoter methylation. *Cell Rep* **3** (2013), pp. 1020-7.

Becker ML, Visser LE, van Schaik RH, Hofman A, Uitterlinden AG & Stricker BH.Interaction between polymorphisms in the OCT1 and MATE1 transporter and metformin response. *Pharmacogenetics and Genomics* 20102038–44. (doi:10.1097/FPC.0b013e328333bb11)

Bolen, S., Feldman, L., Vassy, J., Wilson, L., Yeh, H.C., Marinopoulos, S., Wiley, C., Selvin, E., Wilson, R., Bass, E.B. and Brancati, F.L. Systematic review: comparative effectiveness and safety of oral medications for type 2 diabetes mellitus. *Ann Intern Med* **147** (2007), pp. 386-99.

Buse, J.B., DeFronzo, R.A., Rosenstock, J., Kim, T., Burns, C., Skare, S., Baron, A. and Fineman, M. The Primary Glucose-Lowering Effect of Metformin Resides in the Gut, Not the Circulation: Results From Short-term Pharmacokinetic and 12-Week Dose-Ranging Studies. *Diabetes Care* **39** (2016), pp. 198-205.

Canadian Diabetes Association Clinical Practice Guidelines Expert Committee Canadian Diabetes Association 2013 Clinical

Canto, C., Gerhart-Hines, Z., Feige, J.N., Lagouge, M., Noriega, L., Milne, J.C., Elliott, P.J., Puigserver, P. and Auwerx, J. AMPK regulates energy expenditure by modulating NAD+ metabolism and SIRT1 activity. *Nature* **458** (2009), pp. 1056-60.

Cao, J., Meng, S., Chang, E., Beckwith-Fickas, K., Xiong, L., Cole, R.N., Radovick, S., Wondisford, F.E. and He, L. Low concentrations of metformin suppress glucose production in hepatocytes through AMP-activated protein kinase (AMPK). *J Biol Chem* **289** (2014), pp. 20435-46.

Carbonero, F., Benefiel, A.C. and Gaskins, H.R. Contributions of the microbial hydrogen economy to colonic homeostasis. *Nat Rev Gastroenterol Hepatol* **9** (2012), pp. 504-18.

Carter, D., Howlett, H.C., Wiernsperger, N.F. and Bailey, C.J. Differential effects of metformin on bile salt absorption from the jejunum and ileum. *Diabetes Obes Metab* **5** (2003), pp. 120-5.

Claesson MJ, Cusack S, O'Sullivan O, Greene-Diniz R, de Weerd H, Flannery E, Marchesi JR, Falush D, Dinan T, Fitzgerald G et al: Composition, variability, and temporal stability of the intestinal microbiota of the elderly. *Proc Natl Acad Sci U S A*, 108 (2011) Suppl 1:4586-4591.

Coppedè, F. (2014). Advances in the genetics and epigenetics of neurodegenerative diseases. *Epigenetics Neurodegener. Dis.* 1, 3–31. doi: 10.2478/end-2012-0002

Dayeh, T., Volkov, P., Salo, S., Hall, E., Nilsson, E., Olsson, A.H., Kirkpatrick, C.L., Wollheim, C.B., Eliasson, L., Ronn, T., Bacos, K. and Ling, C. Genome-wide DNA methylation analysis of human pancreatic islets from type 2 diabetic and non-diabetic donors identifies candidate genes that influence insulin secretion. *PLoS Genet* **10** (2014), p. e1004160.

DeFronzo, R.A., Buse, J.B., Kim, T., Burns, C., Skare, S., Baron, A. and Fineman, M. Once-daily delayed-release metformin lowers plasma glucose and enhances fasting and postprandial GLP-1 and PYY: results from two randomised trials. *Diabetologia* **59** (2016), pp. 1645-54.

de la Cuesta-Zuluaga, J., Mueller, N.T., Corrales-Agudelo, V., Velasquez-Mejia, E.P., Carmona, J.A., Abad, J.M. and Escobar, J.S. Metformin Is Associated With Higher Relative Abundance of Mucin-Degrading Akkermansia muciniphila and Several Short-Chain Fatty Acid-Producing Microbiota in the Gut. *Diabetes Care* **40** (2017), pp. 54-62.

De Vadder, F., Kovatcheva-Datchary, P., Goncalves, D., Vinera, J., Zitoun, C., Duchampt, A., Backhed, F. and Mithieux, G. Microbiota-generated metabolites promote metabolic benefits via gut-brain neural circuits. *Cell* **156** (2014), pp. 84-96.

Diamanti-Kandarakis, E., Christakou, C.D., Kandaraki, E. and Economou, F.N. Metformin: an old medication of new fashion: evolving new molecular mechanisms and clinical implications in polycystic ovary syndrome. *Eur J Endocrinol* **162** (2010), pp. 193-212.

Dujic, T., Zhou, K., Yee, S.W., van Leeuwen, N., de Keyser, C.E., Javorsky, M., Goswami, S., Zaharenko, L., Christensen, M.M., Out, M., Tavendale, R., Kubo, M., Hedderson, M.M., van der Heijden, A.A., Klimcakova, L., Pirags, V., Kooy, A., Brosen, K., Klovins, J., Semiz, S., Tkac, I., Stricker, B.H., Palmer, C.N., t Hart, L.M., Giacomini, K.M. and Pearson, E.R. Variants in Pharmacokinetic Transporters and Glycaemic Response to Metformin: A MetGen Meta-Analysis. *Clin Pharmacol Ther* (2016).

Forslund, K., Hildebrand, F., Nielsen, T., Falony, G., Le Chatelier, E., Sunagawa, S., Prifti, E., Vieira-Silva, S., Gudmundsdottir, V., Krogh Pedersen, H., Arumugam, M., Kristiansen, K., Voigt, A.Y., Vestergaard, H., Hercog, R., Igor Costea, P., Kultima, J.R., Li, J., Jorgensen, T., Levenez, F., Dore, J., Nielsen, H.B., Brunak, S., Raes, J., Hansen, T., Wang, J., Ehrlich, S.D., Bork, P. and Pedersen, O. Disentangling type 2 diabetes and metformin treatment signatures in the human gut microbiota. *Nature* **528** (2015), pp. 262-6.

Fung, C.S., Wan, E.Y., Wong, C.K., Jiao, F. and Chan, A.K. Effect of metformin monotherapy on cardiovascular diseases and mortality: a retrospective cohort study on Chinese type 2 diabetes mellitus patients. *Cardiovasc Diabetol* **14** (2015), p. 137.

Gilbert, J.A., Quinn, R.A., Debelius, J., Xu, Z.Z., Morton, J., Garg, N., Jansson, J.K., Dorrestein, P.C. and Knight, R. Microbiome-wide association studies link dynamic microbial consortia to disease. *Nature* **535** (2016), pp. 94-103.

Gong L., Goswami S., Giacomini K. M., Altman R. B., Klein T. E. Metformin pathways: pharmacokinetics and pharmacodynamics. *Pharmacogenetics and Genomics*, 22(2012), pp. 820-7.

Graham, G.G., Punt, J., Arora, M., Day, R.O., Doogue, M.P., Duong, J.K., Furlong, T.J., Greenfield, J.R., Greenup, L.C., Kirkpatrick, C.M., Ray, J.E., Timmins, P. and Williams, K.M. Clinical pharmacokinetics of metformin. *Clin Pharmacokinet* **50** (2011), pp. 81-98.

He R., Zhang D., Lu W., Zheng T., Wan L., Liu F., Jia W. SLC47A1 gene rs2289669 G>A; A variants enhance the glucose-lowering effect of metformin via delaying its excretion in Chinese type 2 diabetes patients. *Diabetes Res Clin Pract*. 109 (2015), pp. 57–63.

Hostalek, U., Gwilt, M. and Hildemann, S. Therapeutic Use of Metformin in Prediabetes and Diabetes Prevention. *Drugs* **75** (2015), pp. 1071-94.

Hullar, M.A. and Fu, B.C. Diet, the gut microbiome, and epigenetics. *Cancer J* **20** (2014), pp. 170-5.

Hundal, R.S., Krssak, M., Dufour, S., Laurent, D., Lebon, V., Chandramouli, V., Inzucchi, S.E., Schumann, W.C., Petersen, K.F., Landau, B.R. and Shulman, G.I. Mechanism by which metformin reduces glucose production in type 2 diabetes. *Diabetes* **49** (2000), pp. 2063-9.

Hur, K.Y. and Lee, M.S. New mechanisms of metformin action: Focusing on mitochondria and the gut. *J Diabetes Investig* **6** (2015), pp. 600-9.

International Diabetes Federation Guideline Development Group Global guideline for type 2 diabetes. *Diabetes Res Clin Pract* **104** (2014), pp. 1-52.

Inzucchi, S.E., Bergenstal, R.M., Buse, J.B., Diamant, M., Ferrannini, E., Nauck, M., Peters, A.L., Tsapas, A., Wender, R. and Matthews, D.R. Management of hyperglycemia in type 2 diabetes, 2015: a patient-centered approach: update to a position statement of the American Diabetes Association and the European Association for the Study of Diabetes. *Diabetes Care* **38** (2015), pp. 140-9.

Jacobsen, S.C., Brons, C., Bork-Jensen, J., Ribel-Madsen, R., Yang, B., Lara, E., Hall, E., Calvanese, V., Nilsson, E., Jorgensen, S.W., Mandrup, S., Ling, C., Fernandez, A.F., Fraga, M.F., Poulsen, P. and Vaag, A. Effects of short-term high-fat overfeeding on genome-wide DNA methylation in the skeletal muscle of healthy young men. *Diabetologia* **55** (2012), pp. 3341-9.

Lan, F., Cacicedo, J.M., Ruderman, N. and Ido, Y. SIRT1 modulation of the acetylation status, cytosolic localization, and activity of LKB1. Possible role in AMP-activated protein kinase activation. *J Biol Chem* **283** (2008), pp. 27628-35.

Lee, J.O., Lee, S.K., Kim, J.H., Kim, N., You, G.Y., Moon, J.W., Kim, S.J., Park, S.H. and Kim, H.S. Metformin regulates glucose transporter 4 (GLUT4) translocation through AMP-activated protein kinase (AMPK)-mediated Cbl/CAP signaling in 3T3-L1 preadipocyte cells. *J Biol Chem* **287** (2012), pp. 44121-9.

Liang, Y.C., Liu, H.J., Chen, S.H., Chen, C.C., Chou, L.S. and Tsai, L.H. Effect of lipopolysaccharide on diarrhea and gastrointestinal transit in mice: roles of nitric oxide and prostaglandin E2. *World J Gastroenterol* **11** (2005), pp. 357-61.

Madiraju, A.K., Erion, D.M., Rahimi, Y., Zhang, X.M., Braddock, D.T., Albright, R.A., Prigaro, B.J., Wood, J.L., Bhanot, S., MacDonald, M.J., Jurczak, M.J., Camporez, J.P., Lee, H.Y., Cline, G.W., Samuel, V.T., Kibbey, R.G. and Shulman, G.I. Metformin suppresses gluconeogenesis by inhibiting mitochondrial glycerophosphate dehydrogenase. *Nature* **510** (2014), pp. 542-6.

Martinez, J.A., Milagro, F.I., Claycombe, K.J. and Schalinske, K.L. Epigenetics in adipose tissue, obesity, weight loss, and diabetes. *Adv Nutr* **5** (2014), pp. 71-81.

McCreight, L.J., Bailey, C.J. and Pearson, E.R. Metformin and the gastrointestinal tract. *Diabetologia* **59** (2016), pp. 426-35.

Mikeska T, Craig JM. DNA methylation biomarkers: cancer and beyond. Genes (Basel). 2014;5(3) 821-864. doi:10.3390/genes5030821. PMID: 25229548; PMCID: PMC4198933.Niu et al. 2016

Musi, N., Hirshman, M.F., Nygren, J., Svanfeldt, M., Bavenholm, P., Rooyackers, O., Zhou, G., Williamson, J.M., Ljunqvist, O., Efendic, S., Moller, D.E., Thorell, A. and Goodyear, L.J. Metformin increases AMP-activated protein kinase activity in skeletal muscle of subjects with type 2 diabetes. *Diabetes* **51** (2002), pp. 2074-81.

Nasri H., and Rafieian-Kopaei M. Metformin: Current knowledge. Journal of Research in Medical Sciences : *The Official Journal of Isfahan University of Medical Sciences*, 19(2014), pp. 658–664.

Nilsson, E., Jansson, P.A., Perfilyev, A., Volkov, P., Pedersen, M., Svensson, M.K., Poulsen, P., Ribel-Madsen, R., Pedersen, N.L., Almgren, P., Fadista, J., Ronn, T., Klarlund Pedersen, B., Scheele, C., Vaag, A. and Ling,

C. Altered DNA methylation and differential expression of genes influencing metabolism and inflammation in adipose tissue from subjects with type 2 diabetes. *Diabetes* **63** (2014), pp. 2962-76.

Petersen, I., Douglas, I. and Whitaker, H. Self controlled case series methods: an alternative to standard epidemiological study designs. *BMJ* **354** (2016), p. i4515.

Ronn, T. and Ling, C. DNA methylation as a diagnostic and therapeutic target in the battle against Type 2 diabetes. *Epigenomics* **7** (2015), pp. 451-60.

Scarpello, J.H., Hodgson, E. and Howlett, H.C. Effect of metformin on bile salt circulation and intestinal motility in type 2 diabetes mellitus. *Diabet Med* **15** (1998), pp. 651-6.

Shin, N.R., Lee, J.C., Lee, H.Y., Kim, M.S., Whon, T.W., Lee, M.S. and Bae, J.W. An increase in the Akkermansia spp. population induced by metformin treatment improves glucose homeostasis in diet-induced obese mice. *Gut* **63** (2014), pp. 727-35.

Song, I. , Shin, H. , Shim, E. , Jung, I. , Kim, W. , Shon, J. and Shin, J. (2008), Genetic Variants of the Organic Cation Transporter 2 Influence the Disposition of Metformin. Clinical Pharmacology & Therapeutics, 84: 559-562. doi:10.1038/clpt.2008.61

Stenman, L.K., Waget, A., Garret, C., Briand, F., Burcelin, R., Sulpice, T. and Lahtinen, S. Probiotic B420 and prebiotic polydextrose improve efficacy of antidiabetic drugs in mice. *Diabetol Metab Syndr* **7** (2015), p. 75.

Jakab Z., WHO Regional Director for Europe, Delivering for diabetes in Europe, 8 December 2010, Brussels, Belgium

The Human Microbiome Project Consortium Structure, function and diversity of the healthy human microbiome. *Nature* **486** (2012), pp. 207-14.

Toperoff, G., Aran, D., Kark, J.D., Rosenberg, M., Dubnikov, T., Nissan, B., Wainstein, J., Friedlander, Y., Levy-Lahad, E., Glaser, B. and Hellman, A. Genome-wide survey reveals predisposing diabetes type 2-related DNA methylation variations in human peripheral blood. *Hum Mol Genet* **21** (2012), pp. 371-83.

Uriarte, G., Paternain, L., Milagro, F.I., Martinez, J.A. and Campion, J. Shifting to a control diet after a high-fat, high-sucrose diet intake induces epigenetic changes in retroperitoneal adipocytes of Wistar rats. *J Physiol Biochem* **69** (2013), pp. 601-11.

Wu, T., Xie, C., Wu, H., Jones, K.L., Horowitz, M. and Rayner, C.K. Metformin reduces the rate of small intestinal glucose absorption in type 2 diabetes. *Diabetes Obes Metab* **19** (2016), pp. 290-293.

Xu, H., Zhou, Y., Liu, Y., Ping, J., Shou, Q., Chen, F. and Ruo, R. Metformin improves hepatic IRS2/PI3K/Akt signaling in insulin-resistant rats of NASH and cirrhosis. *J Endocrinol* **229** (2016), pp. 133-44.

Yuan, W., Xia, Y., Bell, C.G., Yet, I., Ferreira, T., Ward, K.J., Gao, F., Loomis, A.K., Hyde, C.L., Wu, H., Lu, H., Liu, Y., Small, K.S., Vinuela, A., Morris, A.P., Berdasco, M., Esteller, M., Brosnan, M.J., Deloukas, P., McCarthy, M.I., John, S.L., Bell, J.T., Wang, J. and Spector, T.D. An integrated epigenomic analysis for type 2 diabetes susceptibility loci in monozygotic twins. *Nat Commun* **5** (2014), p. 5719.

Zaharenko, Linda & Kalnina, Ineta & Geldnere, Kristine & Bumbure, Alda & Ritenberga, Rota & Nikitina-Zake, Liene & Fridmanis, Davids & Vaivade, Iveta & Pirags, Valdis & Klovins, Janis. (2012). Association of genetic variation in the organic cation transporters OCT1, OCT2 and multidrug and toxin extrusion 1 transporter protein genes with the gastrointestinal side effects and lower BMI in metformin-treated type 2 diabetes patients. Pharmacogenetics and genomics. 22. 659-66. 10.1097/FPC.0b013e3283561666.

Zapata HJ, Quagliarello VJ: The microbiota and microbiome in aging: potential implications in health and age-related diseases. *J Am Geriatr Soc*, 63 (2015), pp. 776-781.

Zhang, X., Zhao, Y., Xu, J., Xue, Z., Zhang, M., Pang, X. and Zhao, L. Modulation of gut microbiota by berberine and metformin during the treatment of high-fat diet-induced obesity in rats. *Sci Rep* **5** (2015), p. 14405.

Zhao, S., Liu, W., Wang, J., Shi, J., Sun, Y., Wang, W., Ning, G., Liu, R. and Hong, J. Akkermansia muciniphila improves metabolic profiles by reducing inflammation in chow diet-fed mice. *J Mol Endocrinol* **58** (2016), pp. 1-14.

Zhong, T, Men, Y, Lu, L, Geng, T, Zhou, J, Mitsuhashi, A, Shozu, M, Maihle, NJ, Carmichael, GG, Taylor, HS & Huang, Y 2017, 'Metformin alters DNA methylation genome-wide via the H19/SAHH axis' *Oncogene*, vol. 36, no. 17, pp. 2345-2354. https://doi.org/10.1038/onc.2016.391

Zhou, G., Myers, R., Li, Y., Chen, Y., Shen, X., Fenyk-Melody, J., Wu, M., Ventre, J., Doebber, T., Fujii, N., Musi, N., Hirshman, M.F., Goodyear, L.J. and Moller, D.E. Role of AMP-activated protein kinase in mechanism of metformin action. *J Clin Invest* **108** (2001), pp. 1167-74.
