# Supplementary figures and images for: Metformin strongly affects transcriptome of peripheral blood cells in healthy individuals
Source: PLoS One. 2019 Nov 8;14(11):e0224835. doi: 10.1371/journal.pone.0224835 (PMC6839856; doi:10.1371/journal.pone.0224835)

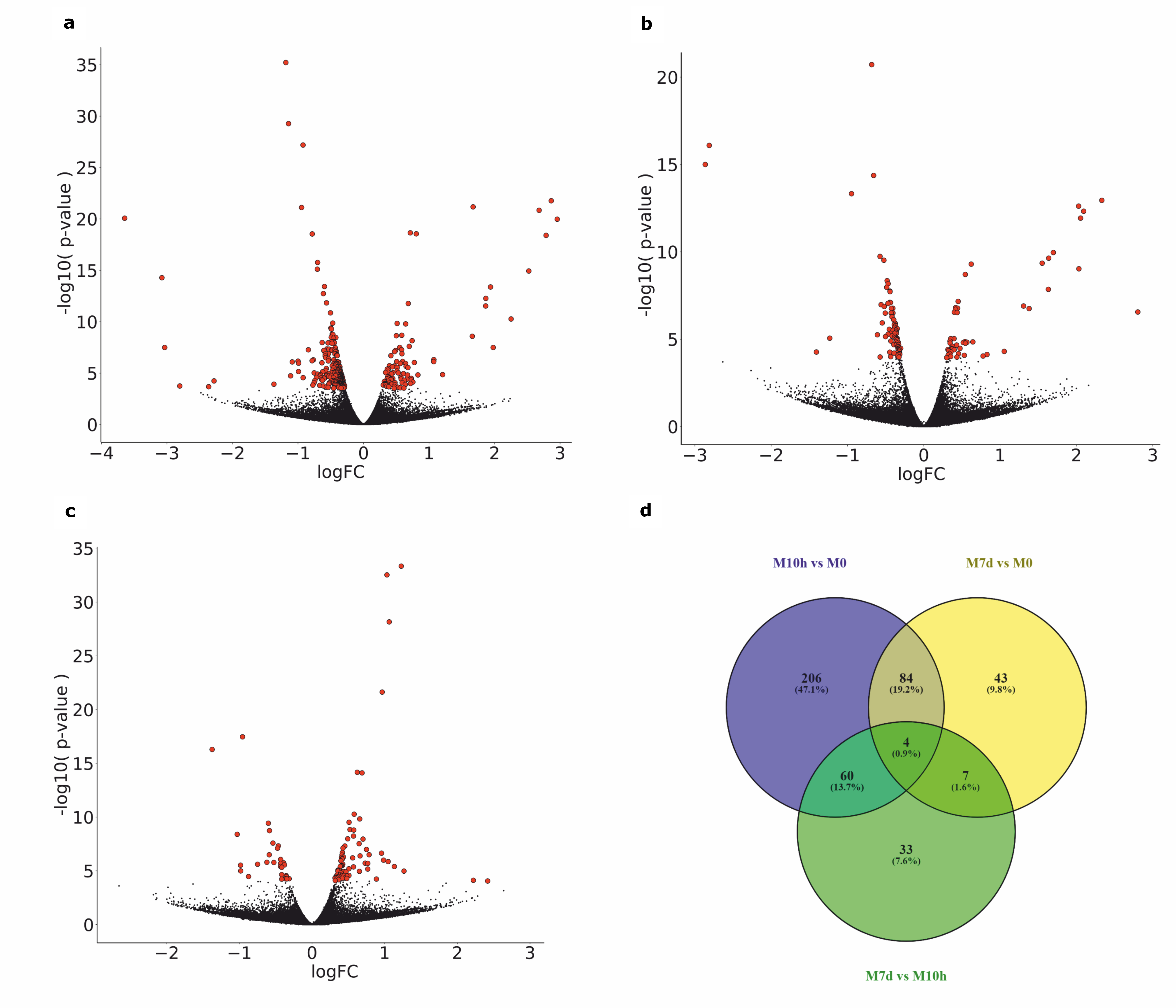

Supplement: S1 Fig — Volcano plots showing the distribution of gene expression in the analyzed contrasts: (A)—M10h vs M0, (B)—M7d vs M0 and (C)—M7d vs M10h. Significance versus log2 fold change is plotted on the y and x axes, respectively. Red dots represent the significant DEGs (FDR < 0.05), black dots—nonsignificant genes. (D)—Venn diagram representing the number of total and overlapping significant DEGs in the analyzed contrasts, DEGs are obtained in the edgeR-sensitive analysis. (TIFF) [file pone.0224835.s007.tiff]

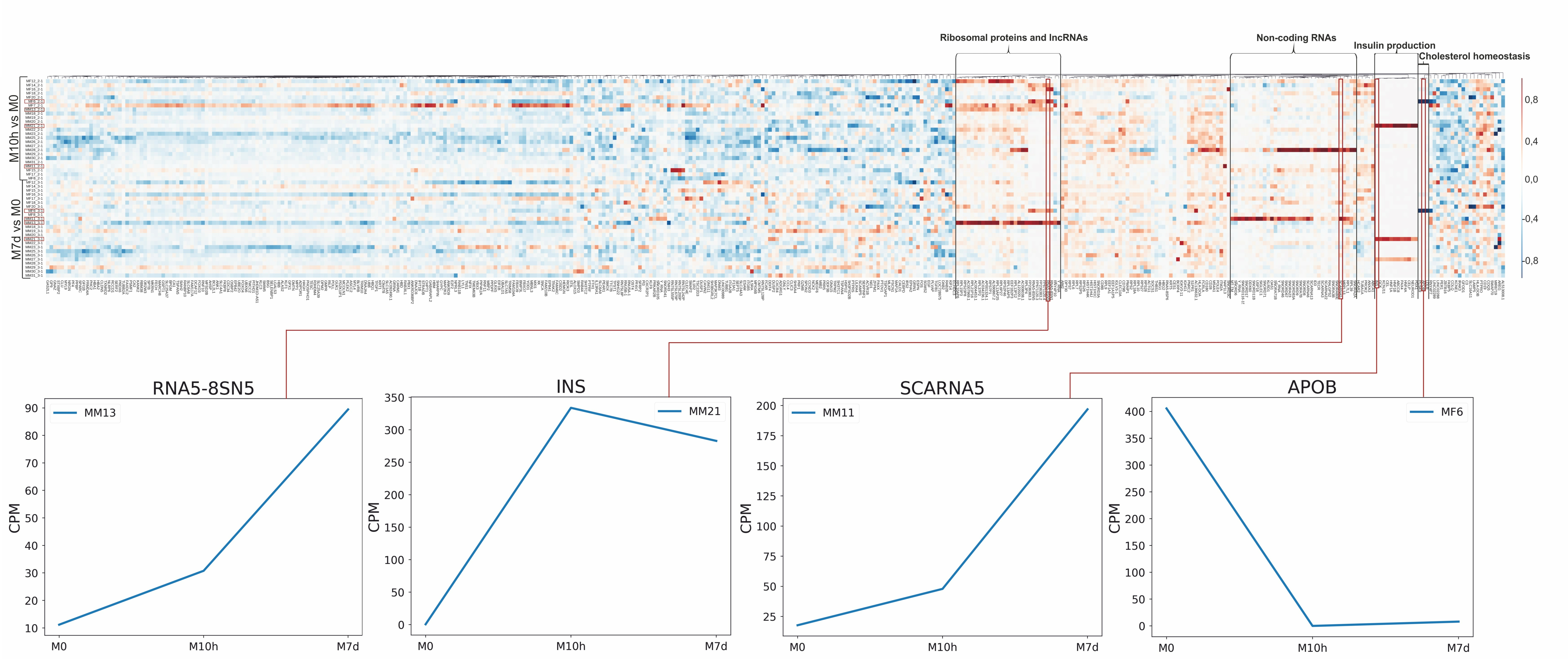

Supplement: S2 Fig — Each row corresponds to one subject in the respective contrast and each column represents a DEG. Normalized sequence read counts were rescaled to lie in range [0,1] and further used to estimate the difference between the gene expression levels in two time-points depending on the particular contrast. DEGs with analogous expression values were clustered at the column level. Line plots show the expression levels (read counts per million) of the most representative genes of each subject-specific gene cluster in three blood sample collection time-points of one representative subject. DEGs are obtained in the edgeR-sensitive analysis. (TIFF) [file pone.0224835.s008.tiff]
